# Long-Term Climate Treaties with a Refunding Club

**DOI:** 10.1007/s10640-021-00597-3

**Published:** 2021-08-25

**Authors:** Hans Gersbach, Noemi Hummel, Ralph Winkler

**Affiliations:** 1 CER-ETH – Center of Economic Research, ETH Zurich and CEPR, Zürichbergstrasse 18, 8092 Zurich, Switzerland; 2 CER-ETH Center of Economic Research, ETH Zurich, Zürichbergstrasse 18, 8092 Zurich, Switzerland; 3 Department of Economics and Oeschger Centre for Climate Change Research, University of Bern, Schanzeneckstrasse 1, 3012 Bern, Switzerland

**Keywords:** Climate change mitigation, Refunding club, International agreements, Sustainable climate treaty, Q54, H23, H41

## Abstract

We show that an appropriately-designed “Refunding Club” can simultaneously solve both free-riding problems in mitigating climate change—participating in a coalition with an emission reduction target and enduring voluntary compliance with the target once the coalition has been formed. Countries in the Club pay an initial fee into a fund that is invested in assets. In each period, part of the fund is distributed among the Club members in relation to the emission reductions they have achieved, suitably rescaled by a weighting factor. We show that an appropriate refunding scheme can implement any feasible abatement path a Club wants to implement. The contributions to the initial fund can be used to disentangle efficiency and distributional concerns and/or to make a coalition stable. Making the grand coalition stable in the so-called “modesty approach” requires less than 0.5% of World GDP. Finally, we suggest ways to foster initial participation, to incorporate equity concerns with regard to developing countries, and ways to ease the burden to fill the initial fund.

## Introduction

### Motivation

International treaties on the provision of global public goods have a fundamental free-riding problem: each country’s contribution will benefit all countries in a non-exclusive and non-rival manner. This Prisoner’s Dilemma aspect and the absence of a supranational authority make international coordination crucial and exceptionally difficult to achieve at the same time. Countries may either lack the incentive to sign an agreement and may prefer to benefit from the signatories’ contributions or they may have incentives not to comply with promises made in an agreement.

In long-run problems extending over decades or even centuries, such as mitigating anthropogenic climate change, a second problem arises. Even if the free-riding problem might be solved temporarily, little is achieved if the international community fails to agree on a subsequent agreement when a first agreement has expired. With respect to anthropogenic climate change, this is a recurrent problem. After the first commitment period of the Kyoto Protocol has expired,^1^ the international community has consistently failed to agree on a subsequent international agreement to reduce greenhouse gas emissions, be it in Copenhagen (2009), Cancún (2010), Durban (2011), Doha (2012) or Warsaw (2013). Although in 2015 a new international mechanism to significantly reduce greenhouse gas emissions, the so-called Paris Agreement (UNFCCC 2015), was adopted, as of March 2021 only a minority of countries has submitted long-term low greenhouse gas emission development strategies, which were due by the end of 2020. In addition, many countries fall short to deliver their self-proclaimed “nationally determined contributions”.

### Treaty and Main Insight

We show that an appropriately-designed “Refunding Club” can simultaneously solve both free-riding problems in mitigating climate change—participating in a coalition with an emission reduction target and enduring voluntary compliance with the target once the coalition has been formed. In particular, we propose and analyze climate treaties that involve a long-run refunding scheme (henceforth “RS”) within a Refunding Club. All countries in a coalition of countries forming a Refunding Club pay an initial fee into a fund that is invested in long-run assets. Countries in the Club maintain full sovereignty over the amount of emissions they abate each year and what policy measures they use to do so. At the end of each year, part of the fund is paid out to participating countries in proportion to the relative GHG emission reductions they have achieved in that year, weighted by country-specific factors.

We integrate the Refunding Club into a dynamic model that incorporates important characteristics of anthropogenic climate change. This requires to allow countries to be arbitrarily heterogeneous with respect to damages and abatement technologies together with a arbitrarily long (but finite) time horizon. Moreover, we incorporate latest scientific evidence of the climate change problem (i.e., we use a carbon budget approach). Then, we establish five main insights. First, any feasible abatement path a coalition of countries sets as a goal can be implemented by a suitably chosen RS. That is, once the corresponding initial fund has been established, the RS will ensure that countries comply with the envisioned country-specific abatement paths. The abatement paths could be the globally optimal paths of the grand coalition or more modest abatement paths by any coalition. Second, since in a treaty, voluntary compliance of countries with their abatement paths is independent from their specific contributions to the initial fund, the RS disentangles efficiency and distributional concerns. For instance, a suitably chosen RS cannot only achieve a Pareto improvement over the decentralized solution, but it can achieve any distribution of the cooperation gains through the allocation of contributions to the initial funds across countries in the Refunding Club.

Third, we use an intertemporal extension of the modest international environmental agreement approach developed by Finus and Maus (2008) to characterize stable coalitions and thus to address the initial participation problem. By combining refunding (to solve the compliance problem) and Finus and Maus’ “modesty approach” (to solve the initial participation problem), we can determine for what level of modesty a coalition, and in particular the grand coalition, can be stabilized. Fourth, using a numerical illustration based upon the RICE-2010 model (Nordhaus 2010) with twelve regions in the world, we calculate ballpark estimates for the funds required to implement the modest grand coalition of less than 0.5% per cent of World GDP. Fifth, we suggest ways to foster initial participation, to incorporate equity concerns by differentiating initial fees across countries and to lower the burden for developing countries, and ways to ease the burden of filling the initial fund. Moreover, we outline how sustainable refunding schemes could be implemented in overlapping generation models.

### Model and Main Formal Results

We study a multi-country model with country-specific emissions, abatement cost functions and damage functions.^2^ Our main formal results are as follows: First, for a given coalition of countries, we introduce a RS, characterized by the set of initial fees payable into a fund by each participating country, a weighting scheme with country-specific refund intensities and a set of reimbursements across time. With a RS, a coalition of countries turns into a Refunding Club. We show that initial fees, the weighting scheme and a feasible sequence of refunds can be devised in such a way that the RS implements any feasible abatement path a coalition of countries wants to achieve through a treaty. That is, together with the abatement decisions of countries outside the coalition, the abatement decisions of countries in the coalition constitute a unique subgame perfect equilibrium and coincide with the goal stipulated in the treaty. Marginal deviations of countries in the coalition would reduce abatement costs marginally but this gain is equal to the corresponding reduction of refunds and increase of damages. A special case is the grand coalition and the implementation of the socially optimal abatement levels in each period and each country as a unique subgame perfect equilibrium with a suitably chosen RS.

Second, for any feasible abatement goal set by a coalition in a treaty, there exists a feasible set of initial fees such that the RS implements a Pareto improvement over the decentralized solution for all coalition members. Moreover, if we allow for negative initial fees, the RS can in fact implement any distribution of the cooperation gains in a coalition, This property of RS to disentangle efficiency and distributional concerns is helpful in achieving initial participation. The former is dealt with by the total amount of the initial fees and the refunding formula with the weighting factors. The latter is dealt with by the country-specific initial fees.

Third, by allowing coalitions to internalize only a fraction of the externalities they create, we can examine the stability of coalitions using the modesty approach developed by Finus and Maus (2008). Drawing on the above results, we show that for any coalition, any degree of modesty can be implemented by a suitable RS, i.e., the abatement choices of countries, in the coalition and outside of it, constitute a unique subgame perfect equilibrium.

Besides the analytical result, we illustrate the working and impact of the refunding scheme in a numerical exercise based upon the RICE-2010 model that takes into account heterogeneities across countries.

### Literature

The starting point for our scheme and its analysis is the large body of game-theoretic literature on the formation of international and self-enforcing environmental agreements^3^ as there is no supranational authority to enforce contracts and to ensure participation and compliance during the duration of a treaty. This literature has provided important insights into the potentialities and limitations of international environmental agreements regarding the solution of the dynamic common-pool problem that characterizes climate change, as discussed and surveyed by Bosetti et al. (2009) and Hovi et al. (2013). Hovi et al. (2013) point out that there are three types of enforcement that are crucial for treaties to reduce global emissions substantially: (i) countries must be given incentives for ratification with deep commitment, (ii) those countries that have committed deeply when ratifying should be given incentives to remain within the treaties, and (iii) they should be given incentives to comply with them. Our Refunding Club satisfies all three requirements. First, when a country joins a coalition, it knows that the Refunding Scheme provides strong incentive for itself and the other members of the coalition to reduce greenhouse gas emissions. Second, once countries have joined the coalition, the Refunding Scheme ensures that countries comply with the envisioned abatement objective. Third, once countries have joined the coalition, they have no incentive to exit, as they would loose all claims on future refunds.

The papers most closely related to our paper are Gersbach and Winkler (2007, 2012) and Gerber and Wichardt (2009, 2013), all of which also incorporate refunding schemes. Gerber and Wichardt (2009) analyze a simple two-stage game in which countries in the first stage choose whether to accede to a treaty. Doing so involves a payment into a fund. In the second stage countries decide on emissions. Only if countries choose a particular emission level that is desired from a global perspective (and, in general, not in the best interest of each country alone) they get a refund paid out of the fund. If refunds (and first-stage deposits, respectively) are sufficiently high, all countries choose socially desired emission levels in the second stage. Participation in the first stage is ensured by the rule that the refunding scheme operates only if all countries participate and contribute their respective payments to the fund. If at least one country does not participate, the deposits of all other countries are immediately repaid, no refunding scheme is established and countries are stuck in the non-cooperative equilibrium. Gerber and Wichardt (2013) extend this framework to an intertemporal framework, in which the continuation of the agreement is challenged by re-occurring deposit stages. As in Gerber and Wichardt (2009) the refunding scheme only operates, respectively continues, if all countries pay their deposits.

In Gersbach and Winkler (2007, 2012), we focussed on the second and third enforcement/commitment problem. We also employed a refunding scheme to incentivice countries to increase their levels of emission abatement above the non-cooperative level. In contrast to Gerber and Wichardt (2009, 2013) our refunding scheme did not prescribe a particular abatement, respectively emission level in order to be eligible for a refund, but employed a continuous refunding rule, in which refunds are increasing with emission abatement. In addition, we analyzed to what extent initial deposits could be decreased by surrendering the revenues of climate policies to the fund (in our case the tax revenues from emission taxes).

### Our Contribution

Relative to the literature mentioned above, we make four contributions in this paper. First, we combine different aspects from the previous literature in a novel way: Like in Gerber and Wichardt (2009) we rely solely on initial payments to finance refunds, as countries might be reluctant to surrender tax sovereignty, but we do not assume that refunding collapses if a country does not match precisely a particular emission level. Instead, we rely on a continuously differentiable refunding rule like in Gersbach and Winkler (2007, 2011, 2012). This means that the sustainable treaties advanced in this paper are rule-based treaties, i.e., the treaties neither fix emission targets nor the carbon price. In contrast to Gersbach and Winkler (2007, 2011, 2012), however, we do not rely on revenues from emission taxes or permit auctions to pay for the initial fees since countries should have full sovereignty over their domestic climate policy and its intensity. The central question in our paper is: Can initial payments to a climate fund engineer solutions aspired by a coalition when refunding continuously adjusts to abatement efforts of countries? None of the preceding work has explored this question.

Second, in contrast to the existing literature on refunding schemes, we build a dynamic model that incorporates important characteristics of anthropogenic climate change. This requires to allow countries to be arbitrarily heterogeneous with respect to damages and abatement technologies together with a arbitrarily long (but finite) time horizon. Moreover, we incorporate latest scientific evidence of the climate change problem (i.e., we use a carbon budget approach, see also Sect. 2 for details). The combination of such a model with a continuous refunding rule results in a dynamic game structure, in which the existence and uniqueness of a subgame perfect equilibrium is neither obvious nor trivial to prove.

Third, we also address the first commitment problem. However, unlike Gerber and Wichardt (2009, 2013), we do not believe that the participation problem can be credibly and realistically solved by an initial stage (or re-occurring intermediate participation stages as in Gerber and Wichardt 2013), in which any agreement is abandoned as soon as only one country is not willing to participate. Models with such an initial participation stage are not renegotiation-proof in the sense that if one country is not willing to participate all other countries would be better off by striking an agreement without the deviating country instead of falling back to the perfectly non-cooperative Nash equilibrium. In fact, we interpret the past announcement of the US’ withdrawal from the Paris Agreement and the subsequent declarations of (almost) all remaining countries to nevertheless stick to the agreement as empirical evidence that making each country pivotal will not work. As a consequence, we investigate the refunding scheme independently of the requirement that all countries participate, i.e., it can be applied to any coalition that forms with initial payments for a climate fund.

To analyze participation, we present an intertemporal generalization of the modesty approach by Finus and Maus (2008), which relies on the standard notion of internal and external stability. In fact, our RS poses a suitable microfoundation for the coalition formation framework in general, and the modest coalition formation framework of Finus and Maus (2008) in particular.^4^ In addition, we explore in Sect. 7 ways to ease the participation and financing problem.^5^

Fourth, the match of anthropogenic climate change characteristics and refunding opens up the possibility to assess for the first time the potential of refunding for slowing down climate change. In particular, we analyze a numerical version of our stylized model based on data of the regional disaggregated integrated assessment model RICE-2010 by Nordhaus (2010) and assess the order of magnitude of financial assets that are needed to finance such a refunding scheme. The calibration exercise reveals that making the grand coalition stable requires less than 0.5% of World GDP for the initial fund.

Finally, we also contribute to solving dynamic public goods problems. At least since Fershtman and Nitzan (1991) it is know that dynamic good problems pose more severe challenges than their static counterparts.^6^ We examine the most severe case when countries cannot commit to any future emission reductions, as no international authority can enforce an agreement on such reductions. The dynamic public good problem is thus particularly severe. The treaties we advance in this paper essentially reduce the public good problem over an infinite horizon to a static problem in which counties are asked to contribute in the initial period to a global fund. Once the global fund has been set up, countries voluntarily choose the desired emission levels in all subsequent periods.^7^

### Organization of the Paper

The paper is organized as follows: in the next section, we set up our model, for which in Sect. 3 we derive the social optimum and the decentralized solution as benchmark cases. The refunding scheme is introduced in Sect. 4, where the existence and uniqueness of RS to implement any solution an arbitrary coalition aspires to is also established. In Sect. 5, we extend the modesty approach to an intertemporal setting and characterize the stability conditions of coalitions with this approach. In Sect. 6 we illustrate our model numerically. In Sect. 7, we discuss practical aspects of the RS, such as initial participation and how to raise initial fees and how sustainable refunding schemes can be implemented in an overlapping generation set-up. Section 8 concludes. Proofs of all propositions are relegated to the “Appendix”.

## The Model

We consider a world with $n \ge 2$ countries characterized by country specific emission functions $E_i$, abatement cost functions $C_i$, and damage functions $D_i$ over a finite (though arbitrarily large) time horizon of $T\ (T > 0)$ running from period $t=0$ to period $t=T$.^8^ Throughout the paper the set of all countries is denoted by $\mathcal {I}$, countries are indexed by *i* and *j*, and time is indexed by *t*.

Emissions of country *i* in period *t* are assumed to equal “business-as-usual” emissions $\epsilon _i$ (i.e., emissions arising if no abatement effort is undertaken) minus emission abatement $a^i_t$:^9^

1 $$\begin{aligned} E_i(a^i_t)=\epsilon _i- a^i_t, \quad i \in \mathcal {I},\quad t=0,\dots ,T\ . \end{aligned}$$

We assume that emission abatement $a^i_t$ is achieved by enacting some national environmental policy, which induces convex abatement costs in country *i*:^10^

2 $$\begin{aligned} C_i(a^i_t) = \frac{\alpha _i}{2} \left( a^i_t\right) ^2\ ,\quad \text {with} \quad \alpha _i>0,\quad i \in \mathcal {I},\quad t=0,\dots ,T\ . \end{aligned}$$

Cumulative global emissions, which are the sum of the emissions of all countries up to period *t*, are denoted by $s_t$:

3 $$\begin{aligned} s_{t+1}=s_{t} + \sum _{i=1}^n E_i(a^i_t)\ ,\quad t=0,\dots ,T, \end{aligned}$$

where the initial stock of cumulative greenhouse gas emissions is denoted by $s_0$.

Recent scientific evidence suggests that global average surface temperature increase is—at least for economically reasonable time scales (i.e., several centuries)—approximately a linear function of cumulative global carbon emissions (see Allen et al. 2009; Matthews et al. 2009; Zickfeld et al. 2009; IPCC 2013). As a consequence, we consider strictly increasing and strictly convex damage costs for each country *i* to depend on cumulative global emissions $s_t$ rather than on atmospheric greenhouse gas concentrations:

4 $$\begin{aligned} D_i(s_t)=\frac{\beta _i}{2}s_t^2\ ,\quad \text {with} \quad \beta _i > 0,\quad i \in \mathcal {I}\ ,\quad t=0,\dots ,T\ . \end{aligned}$$

Countries are assumed to discount outcomes in period *t* with the discount factor $\delta ^t$ with $0<\delta <1$. Finally, we introduce the following abbreviations for later reference:

5 $$\begin{aligned} \mathcal {E} = \sum _{i=1}^n \epsilon _i\ ,\quad \mathcal {A} = \sum _{i=1}^n \frac{1}{\alpha _i},\quad \mathcal {B} = \sum _{i=1}^n \beta _i,\quad \gamma _i = \frac{\beta _i}{\alpha _i},\quad \Gamma = \sum _{i=1}^n \gamma _i \ . \end{aligned}$$

## Decentralized Equilibrium, Global Social Optimum and International Environmental Agreements

Throughout the paper, we assume that a local planner in each country (e.g., a government) seeks to minimize the *total domestic costs*, which—in the absence of any transfers—consist of the discounted sum of domestic abatement and domestic environmental damage costs over all $T + 1$ periods:

6 $$\begin{aligned} K_i = \sum _{t=0}^T \delta ^t \left[ \frac{\alpha _i}{2}\left( a^i_t\right) ^2 + \frac{\beta _i}{2} s_t^2\right] , \qquad i \in \mathcal {I}\ . \end{aligned}$$

We further assume that local planners in all countries have perfect information about the business-as-usual emissions, abatement costs and environmental damage costs of all countries. In addition, in each period *t* local planners in all countries *i* observe the stock of cumulative emissions $s_t$ before they simultaneously decide on the abatement levels $a_t^i$.

Finally, we assume that costs can—at least potentially—be frictionless shared across countries by a transfer scheme $\mathcal {T}$, which is a set of domestic net transfers summing up to zero. Thus, we suppose a *transferable utility* set-up.

### Decentralized Solution and Global Social Optimum

Under these assumptions and in the absence of any international environmental treaty, the decentralized solution is the subgame perfect Nash equilibrium outcome of the game, in which all local planners *i* in period *t* choose abatement levels $a^i_t$ such as to minimize total domestic costs taking the emissions $a^j_t$ of all other countries $j \in \mathcal {I} {\setminus } {i}$ as given.

We solve the game by backward induction, starting from period *T*. It is useful to consider a typical step in this procedure. To this end, suppose that there exists a unique subgame perfect equilibrium for the subgame starting in period $t+1$ with a stock of cumulative greenhouse gas emissions $s_{t+1}$. For the moment, this is assumed to hold in all periods $t+1$ and will be verified in the proof of Proposition 1. Other details of the history of the game apart from the level of cumulative greenhouse gas emissions $s_{t+1}$ do not matter, as only $s_{t+1}$ influences the payoffs of the subgame starting in period $t+1$ and the equilibrium is assumed to be unique.

Given the unique subgame perfect equilibrium for the subgame starting in period $t+1$ with the associated equilibrium payoff $W_{t+1}^i(s_{t+1}),$^11^ country *i*’s best response in period *t*, $\bar{a}_t^i$, is determined by the solution of the optimization problem

7 $$\begin{aligned} V^i_t(s_t)|A_t^{-i} = \max _{a^i_t} \left\{ \delta W^i_{t+1}(s_{t+1})-\frac{\alpha _i}{2}\left( a^i_t\right) ^2- \frac{\beta _i}{2}s_t^2\right\} , \end{aligned}$$

subject to Eq. (3), $W_{T+1}^i(s_{T+1})\equiv 0$, and given the sum of abatement efforts by all other countries $A_t^{-i} = \sum _{j\ne i}a_t^j$. The following proposition establishes the existence and uniqueness of a subgame perfect Nash equilibrium:

#### **Proposition 1**

(Decentralized Solution) *For any time horizon* $T<\infty$, *there exists a unique subgame perfect Nash equilibrium of the game in which all countries non-cooperatively choose domestic abatement levels in every period to minimize the net present value of total domestic costs, characterized by sequences of emission abatements for all countries* *i* *in all periods* *t*, $\left\{ \hat{a}_t^i\right\} _{t=0,\dots ,T}^{i\in \mathcal {I}}$, *and a sequence for the stock of cumulative GHG emissions* $\left\{ \hat{s}_t\right\} _{t=0,\dots ,T}$.

The proof of Proposition 1 is constructive in the sense that we do not only show existence and uniqueness of the subgame perfect equilibrium, but derive closed-form solutions for the corresponding abatement and cumulative GHG emission paths.^12^

In general, the *total global costs*, i.e., the sum of total domestic costs over all countries, are not minimized in the decentralized solution. As a consequence, the decentralized solution is inefficient, as in the global total cost minimum, which is also called the *global social optimum*, all countries could be made better off by an appropriate transfer scheme $\mathcal {T}$, due to the transferable utility assumption.

The reason for the decentralized solution to fall short of the global social optimum is that local planners only take into account the reduction of environmental damages that an additional unit of abatement prevents in their own country and neglect the damage reductions in all other countries. As a consequence, aggregate abatement levels in the decentralized solution are lower compared to the global social optimum and, thus, cumulative greenhouse gas emissions are higher.

The global social optimum is derived by choosing abatement paths $\left\{ a^i_t\right\} _{t=0,\dots ,T}$ for all countries $i \in \mathcal {I}$, such as to minimize the net present value of total global costs consisting of global costs of emission abatement and the sum of domestic environmental damages stemming from the cumulative global emissions:

8 $$\begin{aligned} \min _{\left\{ a^i_t\right\} ^{i\in \mathcal {I}}_{t=0,\dots ,T}} \sum _{t=0}^T \delta ^{t} \sum \limits _{i=1}^{n}\left[ \frac{\alpha _i}{2}\left( a^i_t\right) ^2 + \frac{\beta _i}{2} s_t^2\right] , \end{aligned}$$

There exists a unique global optimum in which the costs of abating an additional marginal unit of emissions have to equal the net present value of all mitigated future damages caused by this additional marginal unit:

#### **Proposition 2**

(Global Social Optimum) *For any time horizon* $T < \infty$ *there exists a unique social global optimum characterized by sequences of emission abatements for all countries* *i* *in all periods* *t*, $\left\{ {a^i_t}^\star \right\} _{t=0,\dots ,T}^{i\in \mathcal {I}}$, *and a sequence for the stock of cumulative greenhouse gas emissions* $\left\{ s_t^\star \right\} _{t=0,\dots ,T}^{i\in \mathcal {I}}$.

Again, we derive closed form solutions for abatement and cumulative emission paths in the global social optimum in the proof of Proposition 2.

### International Environmental Agreement

The inefficiency of the decentralized solution gives incentives to local planners to cooperate in order to reduce total domestic costs. Throughout this paper we refer to these cooperations as *international environmental agreements* or *treaties* for short.^13^ In the framework of our model, the most general definition of an international environmental agreement comprises three components: First, a time horizon *T*, which denotes the duration of the treaty; second, a fixed set $\mathcal {C} \subseteq \mathcal {I}$ of participating countries, also called member countries or simply the *coalition*. Finally, the abatement paths $\left\{ a^i_t\right\} _{t=0,\dots ,T}^{i\in \mathcal {C}}$ of all member countries $i \in \mathcal {C}$, the treaty aspires to implement. We also define aggregated abatement $A^\mathcal {C}_t$ of the coalition $\mathcal {C}$ in period *t* as:

9 $$\begin{aligned} A^\mathcal {C}_t = \sum _{i \in \mathcal {C}} a^i_t, \qquad t=0,\dots ,T\ . \end{aligned}$$

In line with most of the literature on international environmental agreements, we assume that all non-members of the coalition behave as *singletons*, i.e., they non-cooperatively set abatement levels such as to minimize the net present value of their own total domestic costs, as in the decentralized solution, taking the aggregate abatement effort of the coalition and the abatement levels of all other non-member countries as given. Derivation of the subgame perfect equilibrium is analogous to the decentralized solution:

#### **Proposition 3**

(Abatement paths of non-members) *For any time horizon* $T<\infty$ *and any given coalition* $\mathcal {C}$ *with a corresponding sequence of aggregate abatement levels* $\left\{ A^\mathcal {C}_t\right\} _{t=0,\dots ,T}$, *there exists a unique subgame perfect Nash equilibrium of the game in which all non-member countries choose domestic abatement levels in every period* $t=0,\dots ,T$ *to minimize the net present value of total domestic costs, characterized by sequences of emission abatements for all countries* $i \notin \mathcal {C}$ *in all periods* *t*, $\left\{ \check{a}_t^i\right\} _{t=0,\dots ,T}^{i\notin \mathcal {C}}$, *and a sequence for the stock of cumulative GHG emissions* $\left\{ \check{s}_t\right\} _{t=0,\dots ,T}$.

Whether a treaty, as defined above, succeeds in implementing its aspired abatement paths $\left\{ a^i_t\right\} _{t=0,\dots ,T}^{i\in \mathcal {C}}$ mainly depends on two circumstances:

First, the coalition of participating countries has to be *stable* in the sense that no participating country would rather leave the coalition (internal stability) and no non-member country would rather join the coalition (external stability). Whether the conditions of internal and external stability hold, depends on how the aspired abatement paths of the remaining coalition members changed if any of its members would leave the coalition. This question, which set of countries form a stable coalition, is also called the *participation problem*.

Second, even if a treaty is stable in the sense of the participation problem, it still has to make sure that participating countries stick to the aspired abatement paths $\left\{ a^i_t\right\} _{t=0,\dots ,T}^{i\in \mathcal {C}}$. Without any kind of incentive scheme it is, in general, not in the countries’ own best interest to comply with the treaty. Therefore, the question how to incentivize countries to stick to the aspired abatement paths is also called the *compliance problem*.

Most of the literature on international environmental agreements, as reviewed in Sect. 1, has concentrated on the participation problem, while compliance was simply assumed. Although a stable coalition is a sine qua non for a treaty’s success, it is obviously not sufficient, as ample real world examples of non-compliance show. To remedy this shortcoming, we introduce an institutional setting, called a *refunding scheme*, in the next section that implements any feasible abatement paths $\left\{ a^i_t\right\} _{t=0,\dots ,T}^{i\in \mathcal {C}}$, a coalition intends to implement, as the unique subgame perfect Nash equilibrium.

## Refunding Scheme

In the following, we introduce a refunding scheme (RS), a versatile institutional design ensuring the compliance of all members of an international environmental agreement with the aspired abatement paths. The essential idea is that an international fund is established refunding interest earnings to member countries in each period proportionally to their relative emission reductions weighted by country specific refunding weights.

### Rules of the Refunding Scheme

In general, a RS for a given coalition of countries $\mathcal {C}$ and a given time horizon *T* of the treaty is characterized by the set of initial fees $\left\{ f_0^i\right\} ^{i \in \mathcal {C}}$ payable into a global fund by each participating country $i \in \mathcal {C}$, a weighting scheme $\left\{ \lambda _t^i\right\} _{t=0,\dots ,T-1}^{i \in \mathcal {C}}$, and a set of reimbursements $\left\{ R_t\right\} _{t=0,\dots ,T}$. The sequence of events is as follows:

1. At the beginning of period $t=0$ all participating countries pay the initial fees $f_0^i$ into a fund.
2. In every period $t=0,\dots ,T$ all countries $i \in \mathcal {I}$ set abatement levels $a_t^i$.
3. At the end of every period $t=0,\dots ,T$ the RS reimburses the total amount $R_t$ to member countries. In periods $t=0,\dots ,T-1$ each member country $i \in \mathcal {C}$ receives a refund $r^i_t$ that is proportional to the emission reductions they have achieved relative to overall emission abatement of the coalition times a weighting factor $\lambda ^i_t$. In period $t=T$ any remaining fund is repaid in equal shares to all participating countries.

We assume that the assets of the fund are invested at the constant interest rate $\rho$ per period, and the returns add to the global fund in the next period $t+1$. We assume that the interest rate $\rho$ corresponds to the discount factor $\delta$, i.e., $\rho =1/\delta -1$. As the reimbursement $R_t$ is paid to coalition members at the end of each period $t=0,\dots ,T$, the fund at the beginning of period $t+1$ reads

10 $$\begin{aligned} f_{t+1} = (1+\rho )(f_{t}-R_t),\quad t=0,\dots ,T-1, \end{aligned}$$

with an initial fund $f_0=\sum _{i \in \mathcal {C}} f_0^i$. Note that $f_{T+1}=0$, or equivalently $R_T=f_T$.

In addition, the refund $r^i_t$ a member country $i \in \mathcal {C}$ receives in period *t* yields

11 $$\begin{aligned} r^i_t= {\left\{ \begin{array}{ll} \lambda _t^i R_t \frac{a^i_t}{\sum _{j \in \mathcal {C}} a^j_t}, \quad &{}t=0,\dots ,T-1,\\ \frac{R_t}{|\mathcal {C}|},\quad &{}t=T, \end{array}\right. } \end{aligned}$$

with a weighting scheme satisfying

12 $$\begin{aligned} \sum _{i \in \mathcal {C}} \lambda _t^i \frac{a^i_t}{\sum _{j \in \mathcal {C}} a^j_t} =1,\quad t=0,\dots ,T-1\ . \end{aligned}$$

The weighting scheme accounts for the fact that countries are heterogeneous with respect to business-as-usual emissions, abatement costs and environmental damage costs.

### Existence and Uniqueness of the Refunding Scheme

In the following, we show that for any given treaty, characterized by a time horizon *T*, a coalition $\mathcal {C}$ and feasible coalition abatement paths $\left\{ a_t^i\right\} _{t=0,\dots ,T}^{i \in \mathcal {C}}$, there exists a set of initial fees $\left\{ f_0^i\right\} ^{i \in \mathcal {C}}$, a weighting scheme $\left\{ \lambda _t^i\right\} _{t=0,\dots ,T-1}^{i \in \mathcal {C}}$ and refunds $\left\{ R_t\right\} _{t=0,\dots ,T-1}$ such that the RS implements the aspired abatement paths $\left\{ a_t^i\right\} _{t=0,\dots ,T}^{i \in \mathcal {C}}$ of the coalition $\mathcal {C}$ as the unique subgame perfect Nash equilibrium in which all countries set emission abatement levels in all periods to minimize the net present value of their own total domestic costs (which also includes initial payments and refunds for members of the coalition).

To this end, we first define a *feasible coalition abatement path*. A feasible coalition abatement path has the property that it lies in between the abatement paths of the decentralized solution and the social global optimum for all coalition member countries $i \in \mathcal {C}$ and all time periods $t=0,\dots ,T$:^14^

13 $$\begin{aligned} \hat{a}_t^i \le \tilde{a}_t^i \le {a_t^i}^\star ,\qquad i \in \mathcal {C},\quad t=0,\dots ,T\ . \end{aligned}$$

Note that, by construction, all feasible coalition abatement paths obeying conditions (13) are optimal in the last period *T*, as $\tilde{a}_T^i = 0$ for all $i \in \mathcal {C}$. Then, the following Proposition holds:

#### **Proposition 4**

(Existence of the RS) *Given a treaty characterized by the coalition* $\mathcal {C}$, *a time horizon* *T* *and feasible coalition abatement paths* $\left\{ \tilde{a}_t^i\right\} _{t=0,\dots ,T}^{i \in \mathcal {C}}$, *there exist a RS characterized by a set of initial fees* $\left\{ \tilde{f}_0^i\right\} ^{i \in \mathcal {C}}$, *a sequence of feasible refunds* $\left\{ \tilde{R}_t\right\} _{t=0,\dots ,T-1}$, *and a weighting scheme* $\{\tilde{\lambda }_t^i\}_{t=0,\dots ,T-1}^{i\in \mathcal {C}}$ *such that the outcome of the unique subgame perfect Nash equilibrium of the game, in which all countries non-cooperatively choose domestic abatement levels in every period to minimize the net present value of total domestic costs, coincides with the aspired abatement paths* $\left\{ \tilde{a}_t^i\right\} _{t=0,\dots ,T}^{i \in \mathcal {C}}$ for *all member countries* $i \in \mathcal {C}$ *and the abatement paths* $\left\{ \check{a}_t^i\right\} _{t=0,\dots ,T}^{i\notin \mathcal {C}}$, *as given by Proposition* 3, *for all non-member countries* $i \notin \mathcal {C}.$

The idea of the proof is to choose a reward system that renders all member countries’ aspired abatement levels under the RS as best responses to the given abatement levels of all other countries (within and outside the coalition). As shown in the proof of Proposition 4 in the “Appendix”, the RS is characterized by a uniquely determined sequence of refunds $\left\{ \tilde{R}_t\right\} _{t=0,\dots ,T-1}$ and a weighting scheme $\left\{ \tilde{\lambda }_t^i\right\} _{t=0,\dots ,T-1}^{i\in \mathcal {C}}$. Yet, the set of initial fees is not unambiguously determined. In fact, all sets of initial fees, the sum of which exceeds the minimal initial global fund $\tilde{f}_0$ with

14 $$\begin{aligned} \tilde{f}_0 = \sum _{t=0}^{T-1} \frac{\tilde{R}_t}{(1+\rho )^t}, \end{aligned}$$

render a feasible RS that implements the treaty with aspired coalition abatement paths $\left\{ \tilde{a}_t^i\right\} _{t=0,\dots ,T}^{i \in \mathcal {C}}$. The intuition is that the global fund needs the minimum size $\tilde{f}_0$ in order to be able to pay sufficiently high refunds $\tilde{R}_t$ such that countries stick to the aspired abatement levels in all periods. Any excess funds are redistributed in equal shares in the last period, in which the abatement level is zero independently of the refund. Even if we restrict attention to the minimal initial global fund $\tilde{f}_0$, we are free in how to distribute the burden of raising the initial fund across countries.

#### **Proposition 5**

(Uniqueness of the RS) *For a given treaty characterized by the coalition* $\mathcal {C}$, *a time horizon* *T* *and a set of feasible coalition abatement paths* $\left\{ \tilde{a}_t^i\right\} _{t=0,\dots ,T}^{i \in \mathcal {C}}$, *the refunding scheme is only unique with respect to the minimal initial global fund* $\tilde{f}_0$. *In particular, there exists a feasible set of initial fees* $f_0^i$ *satisfying* $\sum _{i \in \mathcal {C}} f_0^i = \tilde{f}_0$ *such that the RS constitutes a Pareto improvement over the decentralized solution for all coalition members* $i \in \mathcal {C}$.

The intuition for this result is that compared to the decentralized solution all countries are better off under the RS if their initial fee was zero. As a consequence, there is a positive initial fee $\hat{f}_0^i$ that would leave country *i* equally well off under the RS compared to the decentralized solution. In the proof of Proposition 5 in the “Appendix”, we show that the sum $\hat{f}_0 =\sum _{i \in \mathcal {C}} \hat{f}_0^i$ exceeds $\tilde{f}_0$. As a consequence, we can set the initial fee below $\hat{f}_0^i$ making all countries better off.^15^

In summary, we have shown that the RS can implement any feasible coalition abatement path and gives ample freedom in how to raise the necessary initial fund. The former feature of the RS may be important, as, in general, international climate policy is not shaped along standard economic cost-benefit analyses such as the derivation of the global social optimum in Sect. 3.1. In fact, the policy goal, for which global consensus is sought after, is to limit greenhouse gas emissions to such an extent that the global mean surface temperature increase is not exceeding 2 $^{\circ }\hbox {C}$ against preindustrial levels (see, for example, EU 2005; UNFCCC 2009, 2015). As the global mean surface temperature increase is predominantly determined by cumulative greenhouse gas emission, such a temperature goal can be translated into a stock of permissible cumulated greenhouse gas emissions, a so-called *global carbon budget*. Starting from the current stock of cumulative greenhouse gas emissions, estimates for this remaining carbon budget—as of beginning of 2018—roughly range between 320 and 555 trillion tonnes of carbon (GtC) (IPCC 2018).^16^ Thus, Proposition 4 says that once the world community has agreed on an abatement path, the RS is able to implement it as a unique subgame perfect Nash equilibrium no matter how ambitious this abatement path is compared to the social global optimum. In particular, the RS is compatible with the idea of “nationally determined contributions”, as detailed in Article 3 of the Paris Agreement (UNFCCC 2015).

The latter feature of the RS implies that it cannot only achieve a Pareto improvement, but can in fact implement any distribution of the cooperation gain, i.e., the difference of the net present value of the total global costs between the decentralized solution and the social global optimum, if we allow initial fees to be negative for at least some countries. This property of the RS to disentangle efficiency and distributional concerns is helpful in achieving initial participation, as we shall discuss in Sect. 7.1.

## The Modesty Approach to Refunding

So far, we have focused on the compliance problem, i.e., how to incentivize member countries to stick to the aspired abatement paths of a given international environmental agreement. Although Proposition 5 has established that all member countries can be made better off under the RS compared to the fully decentralized solution, as characterized by Proposition 1, this does not imply that any treaty is stable in the sense that all member countries have an incentive to join the coalition in the first place.^17^ As already mentioned in Sect. 3.2, it is crucial to characterize how aspired abatement paths changed if any coalition members were to leave the treaty (or, more precisely, not to participate in the treaty in the first place). In the following, we showcase how questions of participation and compliance can be discussed simultaneously by applying the RS, as characterized in Sect. 4, to an intertemporal extension of the modest international environmental agreement approach developed by Finus and Maus (2008).

### An Intertemporal Extension of Modesty

The standard coalition formation game is a two stage game, in which all countries in the first stage simultaneously decide whether to join an international agreement. In the second stage, all countries simultaneously set emission abatement levels. Non-member countries choose abatement levels non-cooperatively by minimizing their own domestic costs, taking the abatement levels of all other countries as given, while coalition members are supposed to choose emissions abatement levels such as to minimize the sum of total domestic costs over all member countries. Finus and Maus (2008) allow for modest international environmental agreements by specifying that member countries only internalize a fraction $\mu \in [0, 1]$ of the externalities within the coalition.

Applying this idea to our intertemporal model framework, results in the following two stage game:

1. At the beginning of period $t=0$ all countries simultaneously decide whether to join an international environmental agreement.
2. In all periods $t=0,\dots ,T$ all countries simultaneously decide on emission abatement levels.
  - Non-member countries choose abatement levels minimizing the net present values of their total domestic costs, taking the abatement levels of the coalition and the other non-members as given, resulting in abatement paths $\left\{ \check{a}_t^i\right\} _{t=0,\dots ,T}^{i \in \mathcal {C}}$ as characterized by Proposition 3.
  - Members of the coalition $\mathcal {C}$ set abatement levels such as to
    15 $$\begin{aligned} \min _{\left\{ a^i_t\right\} ^{i \in \mathcal {C}}_{t=0,\dots ,T}} \sum _{t=0}^T \delta ^{t} \sum _{i \in \mathcal {C}} \left[ \frac{\alpha _i}{2}\left( a^i_t\right) ^2 + \mu \frac{\beta _i}{2} s_t^2\right] , \end{aligned}$$
    taking the emission levels of non-member countries as given.

The parameter $\mu$ in Eq. (15) can be interpreted as the degree of modesty. It can be interpreted as the fraction $\mu$ of externalities the coalition internalizes among its members. This formulation essentially entails a more modest emission reduction goal. The higher $\mu$, the higher the emission abatement goal of the coalition. For $\mu =1$ the treaty internalizes all externalities coalition members impose on each other, which is the assumption of the standard coalition formation set-up.

### Combining Modesty and Refunding

While assuming that the coalition sets abatement levels according to Eq. (15) allows for a parsimonious way to reconcile the empirical observation of “large but modest” agreements with the prediction of the coalition formation framework, one might ask why coalition members should comply with these aspired abatement paths of the treaty, as they are, in general, not in their best interest (in terms of minimizing the net present value of total domestic costs). This is where the RS, as characterized in Sect. 4 comes into play. As we shall proof in Proposition 6, the aspired abatement paths characterized by Eq. (15) constitute feasible coalition abatement paths that can be implemented via an appropriate RS by virtue of Proposition 4. Thus, the RS serves as a microfoundation to implement the aspired abatement paths characterized by the modest coalition formation framework.

As usual, we analyze the intertemporal modest coalition formation game with refunding by backward induction, i.e., we first characterize the subgame perfect Nash equilibrium of the second stage, for given time horizon *T* and given coalition $\mathcal {C}$.

#### **Proposition 6**

(Abatement paths in SPE of second stage) *For any time horizon* $T<\infty$, *any given coalition* $\mathcal {C}$ *and any degree of modesty* $\mu$, *there exists a unique subgame perfect Nash equilibrium of the game in which all non-member countries* $i \notin \mathcal {C}$ *choose domestic abatement levels in every period* $t=0,\dots ,T$ *to minimize the net present value of total domestic costs, and all member countries* $i \in \mathcal {C}$ *set abatement levels according to Eq.* (15). *The subgame perfect Nash equilibrium is characterized by emission abatements paths* $\left\{ \check{a}_t^i\right\} _{t=0,\dots ,T}^{i\notin \mathcal {C}}$ *for all countries* $i \notin \mathcal {C}$ *and* $\left\{ \tilde{a}_t^i\right\} _{t=0,\dots ,T}^{i\in \mathcal {C}}$ *for all countries* $i \in \mathcal {C}$ *and a corresponding path* $\left\{ s_t\right\} _{t=0,\dots ,T}$ *for the stock of cumulative GHG emissions*.

The proof of Proposition 6 in the “Appendix” is constructive, as we derive the unique closed-form solutions of the abatement paths in the subgame perfect Nash equilibrium of the second stage. Moreover, we show that the decentralized solution, as given by Proposition 1, and the global social optimum, as characterized by Proposition 2, are boundary solutions of Proposition 6, which apply in the case that the coalition only consists of at most one member country or all countries are members of the coalition and $\mu =1$. As a consequence, the assumptions of Proposition 4 apply, and any feasible abatement path as defined in Eq. (13) of the modest coalition formation game can be implemented by an appropriate RS.

Having solved the compliance problem in the second stage by employing the RS, we can now turn to the participation problem in the first stage. Anticipating the outcome of the second stage, a coalition is a subgame perfect Nash equilibrium outcome of the first stage, if no country has an incentive to unilaterally change its membership status. Thus, all member countries $i \in \mathcal {C}$ must not be better off if they were not in the coalition, and all non-member countries $i \notin \mathcal {C}$ must be better off than if they were by joining the coalition. If we denote, for any given coalition $\mathcal {C}$ and modesty parameter $\mu$, the net present value of total domestic costs of member countries $i \in \mathcal {C}$ by $\tilde{K}_i(\mathcal {C},\mu )$ and the net present value of total domestic costs of non-member countries $i \notin \mathcal {C}$ by $\check{K}_i(\mathcal {C},\mu )$, then the conditions of internal and external stability read in our transferable utility set-up:^18^

16a $$\begin{aligned}&\sum _{j \in \mathcal {C}} \tilde{K}_j(\mathcal {C},\mu ) - \sum _{j \in \mathcal {C}{\setminus } i} \tilde{K}_j (\mathcal {C}{\setminus } i,\mu ) \le \check{K}_i(\mathcal {C}{\setminus } i,\mu ),\qquad \forall \ i \in \mathcal {C}, \end{aligned}$$

16b $$\begin{aligned}&\sum _{j \in \mathcal {C} \cup i} \tilde{K}_j(\mathcal {C} \cup i,\mu ) - \sum _{j \in \mathcal {C}} \tilde{K}_j(\mathcal {C},\mu ) > \check{K}_i(\mathcal {C},\mu ),\qquad \forall \ i \notin \mathcal {C}\ . \end{aligned}$$

We note that the stability conditions can be formulated without explicitly invoking the RS. The reasons is twofold. First, the RS does not change the sum of the net present value of total domestic costs over all member countries, as the net present value of all refunds is, by construction, equal to the initial fund. Second, according to Proposition 6, there exists an appropriate RS for any coalition structure $\mathcal {C}$ such that it is in the best interest of coalition members to stick to the agreement. Therefore, the conditions of internal and external stability implicitly also involve all elements of the RS in different coalition arrangements $\mathcal {C}, \mathcal {C}{\setminus } i$ and $\mathcal {C} \cup i$, namely how much a country in the coalition has to pay into the initial fund, how much it will abate inside and outside the coalition, how many refunds it will obtain when in the coalition and how many damages occur. Hence, for instance, initial contributions have to be chosen such that stability conditions are met for each individual country.

We note that the refunding approach with its transfers balances asymmetries between countries in a coalition in an optimal way,^19^ i.e., to achieve the abatement objective of the coalition by providing incentives for countries to comply and by making sure that countries want to join the coalition.

Even in the static modest international environmental agreement framework it is not possible to analytically analyze coalition stability for quadratic damage functions and heterogenous countries. As a consequence, we shall concentrate attention to a particularly interesting case, calibrate our model and derive numerical results. The particular question, we want to address is for what level of modesty $\mu$ the grand coalition $\mathcal {C}=\mathcal {I}$ can be stabilized. For the grand coalition, only internal stability (16a) is relevant, which can be re-arranged to yield:

17 $$\begin{aligned} \sum _{j \in \mathcal {I}} \tilde{K}_j(\mathcal {I},\mu ) \le \sum _{i \in \mathcal {I}} \check{K}_i(\mathcal {I} {\setminus } i,\mu )\ . \end{aligned}$$

Thus, the grand coalition is stable if it can guarantee all countries a lower net present value of total domestic costs when they participate in the treaty instead of unilaterally leaving it.

The approach opens up a wide range of further interesting issues, which we leave for future research. For instance, is it globally optimal to stabilize the grand coalition with an appropriate value of $\mu$, instead of being less modest and having only a smaller coalition being stable? Or could better results be achieved by having several smaller regional coalitions with their own abatement objectives and associated refunding schemes?

## Numerical Illustration

To give an idea of the degree of modesty that renders the grand coalition stable and the corresponding order of magnitude needed for the initial fund $f_0$ to implement it via an appropriate RS, we run a numerical exercise. Due to the highly stylized model, the results are rather a numerical illustration than a quantitative analysis.

We follow the RICE-2010 model (Nordhaus 2010) in dividing the world into twelve regions, each of which we assume to act as a “country”, as detailed in Sect. 2.^20^ We also take the “business-as-usual” (BAU) emissions for all twelve regions from Nordhaus (2010). The RICE-2010 model assumes a backstop technology, the price of which decreases over time and fully crowds out fossil fuel based energy technologies by 2265. As a consequence, global CO$_\text {2}$ emissions drop to zero in 2265 in the BAU scenario in which cumulative global CO$_\text {2}$ emissions of 5679.6 GtC have been released into the atmosphere (we assume that cumulative global CO$_\text {2}$ emissions prior to 2015 amount to 550 GtC).

In the global social optimum of the RICE-2010 model, the long-run cumulative global emissions amount to 1470.8 GtC, which implies an increase of the average global surface temperature of approximately 2.9–3 $^{\circ }\hbox {C}$ over preindustrial levels. In addition, carbon neutrality, i.e., zero global GHG emissions are only reached by 2155. In light of the Paris agreement and the recent announcements by the US, EU and China, among other countries, to become carbon neutral by 2050, respectively 2060, the RICE-2010 model’s global social optimum feels somewhat outdated. Unfortunately, there is no updated version of the RICE-2010 model. We deal with this issue in a two step procedure.

**Table 1:** Calibrated abatement and damage cost parameters in tril. USD/GtC$^2$ for all twelve regions

Region	$\alpha _i$	$\beta _i$
US	0.07480	$10.2529 \times 10^{-6}$
EU	0.17741	$11.5362 \times 10^{-6}$
Japan	0.59840	$11.7255 \times 10^{-6}$
Russia	0.28194	$8.34241 \times 10^{-6}$
Eurasia	0.42195	$9.45971 \times 10^{-6}$
China	0.04122	$9.83528 \times 10^{-6}$
India	0.09974	$16.1990 \times 10^{-6}$
MidEast	0.08976	$14.0065 \times 10^{-6}$
Africa	0.17127	$17.4541 \times 10^{-5}$
LatAm	0.16836	$10.3044 \times 10^{-6}$
OHI	0.26429	$11.3390 \times 10^{-6}$
Other	0.08286	$14.1580 \times 10^{-6}$

**Fig. 1:**
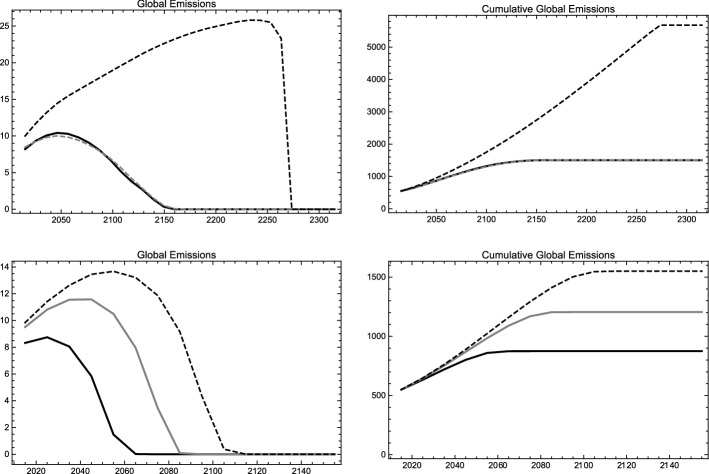
Global emissions (left) and cumulative global emissions (right) in GtC for the RICE-2010 model calibration (upper graphs) and our Paris compatible calibration (lower graphs) in the RICE-2010 BAU scenario (dashed black) and the global social optimum in the RICE-2010 (dashed gray) and our RICE-2010 calibrated model (solid black). The lower graphs show the global social optimum (solid black), the decentralized solution (dashed black) and the stable grand coalition (solid gray) in the Paris compatible calibration

First, we calibrate our model in such a way that the global social optimum in our model resembles the optimal solution of the RICE-2010 model as closely as possible. Therefore, we calibrate the relative damage parameters for each region by fitting quadratic functions to the damage functions used in the RICE-2010 model. Then we re-scale all damage parameters such that damages in the BAU scenario in the year 2095 amount to 12 trillion USD or 2.8% of global output as in Nordhaus (2010, 11723). We calibrate the abatement cost parameters such that the emission paths in the global social optimum of all twelve regions resemble the optimal solution of the RICE-2010 model as closely as possible, under the constraint that the abatement cost parameters decline at a unique and constant rate. Table 1 shows the calibrated abatement and damage cost parameters for all twelve world regions. Abatement cost parameters decrease at the rate of $\xi =1.65\%$ per year, implying a drop of approximately 15.1% per decade.^21^ In line with the RICE-2010 model, we employ a discount rate of 5% per year, which corresponds to a discount factor of $\delta = 0.6139$ for each ten year period. While it is not possible to perfectly mimic the outcome of a sophisticated integrated assessment model as the RICE-2010 model with our simple theoretical model, both global GHG emissions as well as cumulative global emissions match reasonably well (see upper graphs in Fig. 1).

Second, we increase the rate at which the abatement cost parameters decline to $\xi =5\%$ per year implying a decadal drop of 38.6%. Under these conditions, our model calculates a global social optimum in which carbon neutrality is reached by 2065 and cumulative global emissions level off at 874.6 GtC. This corresponds to an average global surface temperature increase between 1.7 and 1.8 $^{\circ }\hbox {C}$, which we consider compatible with the goals of the Paris Agreement. In this “Paris compatible” calibration, the long-run level of cumulative global emissions in the decentralized solution amounts to 1550.1 GtC, which approximately corresponds to a 3.1 $^{\circ }\hbox {C}$ increase of average global surface temperature. In this scenario, carbon neutrality would be achieved by 2115 (see lower graphs in Fig. 1).

Seeking the upper bound of the degree of modesty, for which the grand coalition for the Paris compatible model calibration is just stable, we find $\mu =26.594\%$. In this case, long-run cumulative global emissions amount to 1204.7 GtC, which closes approximately half of the gap between the decentralized solution and the global social optimum. Yet, with a temperature increase of approximately 2.4 $^{\circ }\hbox {C}$, the stable grand coalition would fall short of the 2 $^{\circ }\hbox {C}$ temperature target.

**Table 2:** Net present value of refunds and maximum initial fees $\hat{f}_0^i$ for all 12 regions in tril. USD and % of world GDP for the stable grand coalition in the Paris compatible model calibration (degree of modesty $\mu =26.594\%$)

Region	NPV ref. [tril. USD]	NPV ref. [% WGDP]	$\hat{f}_0^i$ [tril. USD]	$\hat{f}_0^i$ [% WGDP]
US	0.3674	0.0453	0.2926	0.0361
EU	0.1555	0.0192	0.1612	0.0199
Japan	0.0396	0.0049	0.0884	0.0109
Russia	0.0863	0.0107	0.0840	0.0104
Eurasia	0.0578	0.0071	0.0763	0.0094
China	0.7564	0.0934	0.5762	0.0711
India	0.2214	0.0273	0.2782	0.0343
MidEast	0.2727	0.0337	0.2785	0.0344
Africa	0.1147	0.0142	0.2232	0.0275
LatAm	0.1698	0.0210	0.1562	0.0193
OHI	0.0899	0.0111	0.1162	0.0143
Other	0.3068	0.0379	0.3075	0.0379
Sum	2.6384	0.3256	2.6384	0.3256

An initial fund of 2.64 tril. USD or 0.326% of 2015 world GDP is needed to implement the stable grand coalition via a RS. In addition, Table 2 shows the net present value of refunds and the maximum initial fees a region is willing to pay to join the treaty for all 12 regions in tril. USD and % of world GDP. By construction, the sum of initial fees $\hat{f}_0^i$, which makes regions indifferent whether to join the treaty, amounts to the same total of 2.64 tril. USD or 0.326% of 2015 world GDP.

Table 3 shows the development of the refund over time. We observe that total refunds start at very low levels of 19.2 bil. USD per annum corresponding to 0.024% of world GDP, continuously rise until they peak in 2075 at 797 bil. USD per annum corresponding to 0.253% of world GDP. After that they sharply decline and by 2115 no refunds have to be paid anymore, as even in the decentralized solution net zero GHG emissions have been reached. While this general pattern of global refunds is mimicked by the individual regions, there are some differences in magnitude. China receives the highest refunds both in absolute numbers and also as a share of its GDP, peaking in 2075 at 242.03 bil. USD corresponding to 0.546% of its GDP. Africa has the lowest relative peak refunds in 2075 of 34.32 bil. USD or 0.131% of its GDP. The marginal abatement costs start at 49.3 USD per tC (equalling 180.93 USD per ton of CO$_\text {2}$) and rise until 2075, when they peak at 73.6 USD per tC (or 270.11 USD per ton of CO$_\text {2}$).

**Table 3:** Refunds and marginal abatement costs (MAC) over time for the stable grand coalition in the Paris compatible model calibration for all twelve world regions

	2015	2025	2035	2045	2055	2065	2075	2085	2095	2105
*R*(*t*) [tril. USD/a]	0.0192	0.0396	0.0790	0.1512	0.2764	0.4813	0.7970	0.7418	0.3304	0.0474
*R*(*t*) [% World GDP]	0.0237	0.0354	0.0539	0.0817	0.1225	0.1793	0.2526	0.2023	0.0782	0.0098
$\lambda _\text {USA}$	1.07	1.07	1.07	1.07	1.07	1.07	1.07	0.93	0.89	0.57
Refund [bil. USD/a]	2.75	5.67	11.30	21.63	39.55	68.86	114.78	83.59	34.58	3.10
Refund [% GDP]	0.017	0.027	0.044	0.071	0.112	0.170	0.252	0.164	0.061	0.005
MAC [USD/tC]	49.3	55.4	61.3	66.5	70.4	72.8	73.6	53.4	33.4	20.9
$\lambda _\text {EU}$	0.93	0.93	0.93	0.93	0.93	0.93	0.94	1.34	1.65	3.85
Refund[bil. USD/a]	1.02	2.09	4.17	7.99	14.61	25.43	42.38	70.12	39.66	13.00
Refund [% GDP]	0.006	0.010	0.017	0.027	0.043	0.067	0.101	0.153	0.079	0.024
MAC [USD/tC]	49.3	55.4	61.3	66.5	70.4	72.8	73.6	73.7	49.0	30.7
$\lambda _\text {Japan}$	0.89	0.89	0.89	0.89	0.89	0.89	0.89	0.95	0.82	0.02
Refund [bil. USD/a]	0.29	0.59	1.18	2.26	4.13	7.18	11.97	12.31	4.41	0.02
Refund [% GDP]	0.006	0.011	0.020	0.036	0.061	0.097	0.150	0.143	0.048	0.0002
MAC Japan [USD/tC]	49.3	55.4	61.3	66.5	70.4	72.8	73.6	61.3	36.7	22.5
$\lambda _\text {Russia}$	1.02	1.02	1.02	1.02	1.02	1.02	0.91	0.62	0.52	0
Refund [bil. USD/a]	0.70	1.44	2.87	5.50	10.06	17.51	23.88	11.41	4.00	0
Refund [% GDP]	0.031	0.052	0.089	0.149	0.241	0.377	0.467	0.205	0.067	0
MAC Russia [USD/tC]	49.3	55.4	61.3	66.5	70.4	72.8	67.6	41.1	24.9	15.5
$\lambda _\text {Eurasia}$	0.97	0.97	0.97	0.97	0.97	0.97	0.98	0.70	0.64	0.13
Refund [bil. USD/a]	0.44	0.92	1.83	3.50	6.39	11.13	18.55	9.86	3.85	0.11
Refund [% GDP]	0.041	0.060	0.090	0.136	0.203	0.296	0.419	0.193	0.066	0.002
MAC [USD/tC]	49.3	55.4	61.3	66.5	70.4	72.8	73.6	46.8	29.2	18.4
$\lambda _\text {China}$	1.24	1.24	1.24	1.24	1.24	1.24	1.24	0.91	0.79	0
Refund [bil. USD/a]	5.79	11.95	23.82	45.59	83.34	145.12	242.03	137.68	50.27	0
Refund [% GDP]	0.049	0.071	0.109	0.167	0.255	0.378	0.546	0.273	0.089	0
MAC [USD/tC]	49.3	55.4	61.3	66.5	70.4	72.8	73.6	49.1	30.1	18.7
$\lambda _\text {India}$	0.81	0.81	0.81	0.81	0.81	0.81	0.81	0.95	0.79	0
Refund [bil. USD/a]	1.57	3.23	6.44	12.33	22.53	39.23	65.39	78.93	29.69	0
Refund [% GDP]	0.034	0.044	0.059	0.081	0.113	0.157	0.212	0.211	0.067	0
MAC [USD/tC]	49.3	55.4	61.3	66.5	70.4	72.8	73.6	65.9	43.3	28.2
$\lambda _\text {MidEast}$	0.90	0.90	0.90	0.90	0.90	0.90	0.90	1.01	1.01	0.27
Refund [bil. USD/a]	1.94	3.99	7.96	15.24	27.85	48.49	80.83	91.09	41.44	1.58
Refund [% GDP]	0.034	0.046	0.065	0.092	0.134	0.190	0.262	0.248	0.095	0.003
MAC [USD/tC]	49.3	55.4	61.3	66.5	70.4	72.8	73.6	63.8	42.3	27.4
$\lambda _\text {Africa}$	0.73	0.73	0.73	0.73	0.73	0.73	0.73	0.79	0.66	0
Refund [bil. USD/a]	0.82	1.70	3.38	6.47	11.83	20.60	34.32	37.19	14.77	0
Refund [% GDP]	0.034	0.039	0.048	0.060	0.078	0.102	0.131	0.111	0.035	0
MAC [USD/tC]	49.3	55.4	61.3	66.5	70.4	72.8	73.6	64.0	44.2	29.5
$\lambda _\text {LatAm}$	0.98	0.98	0.98	0.98	0.98	0.98	0.98	1.35	1.66	4.75
Refund [bil. USD/a]	1.12	2.31	4.61	8.83	16.15	28.12	46.86	72.51	40.11	16.71
Refund [% GDP]	0.016	0.022	0.033	0.049	0.072	0.105	0.149	0.198	0.095	0.035
MAC [USD/tC]	49.3	55.4	61.3	66.5	70.4	72.8	73.6	71.7	46.7	30.4
$\lambda _\text {OHI}$	0.92	0.92	0.92	0.92	0.92	0.92	0.93	0.82	0.65	0
Refund [bil. USD/a]	0.67	1.39	2.77	5.30	9.69	16.86	28.10	21.24	7.08	0
Refund [% GDP]	0.013	0.021	0.035	0.056	0.091	0.143	0.218	0.151	0.047	0
MAC [USD/tC]	49.3	55.4	61.3	66.5	70.4	72.8	73.6	54.4	32.8	20.1
$\lambda _\text {Other}$	0.91	0.91	0.91	0.91	0.91	0.91	0.91	1.13	1.25	1.78
Refund [bil. USD/a]	2.11	4.34	8.66	16.58	30.30	52.76	87.95	115.91	60.50	12.87
Refund % GDP]	0.051	0.063	0.082	0.109	0.147	0.198	0.261	0.277	0.119	0.021
MAC [USD/tC]	49.3	55.4	61.3	66.5	70.4	72.8	73.6	67.4	45.9	30.8

In summary, the refunding scheme can stabilize a grand coalition that bridges half the gap between the global social optimum and the decentralized solution in our Paris compatible model calibration. While the net present value of funds needed is sizeable at 2.64 tril. USD, it is not out of reach, when compared to other funds raised in situations of global crisis, such as the latest global financial crisis or the Corona pandemic. We would like to stress that our quantitative exercise is only an illustrative example to gauge the order of magnitude of what an initial fund would look like.

## Discussion

So far we have focused first on how a refunding scheme can implement any goal a coalition has set and second, on how coalition stability can be achieved, and in particular the stability of the grand coalition. We have seen that a refunding scheme transforms the intertemporal climate-policy problem into a standard, static public-goods problem. Once all countries in the stable coalition have made their initial contribution, and have agreed on the refunding parameters, countries follow the envisioned path of abatement voluntarily and would be worse off by forfeiting refunds.

Numerous further issues are relevant for the design of refunding scheme and the use of Refunding Clubs. We address them in this section, as they deserve further scrutiny.

### Increasing Initial Fees and a Refunding Club

At the initial level, when countries are pondering whether to sign the treaty and to pay the initial fee, the free-rider problem is present. If all other countries participate and if aggregate initial fees are high, this country would benefit from all other countries’ abatement efforts, without having to pay the initial fee and to compete for refunds. Hence, the question is how better solutions than the one induced by the modesty approach could be achieved.

The ideal solution lies in making countries—and large countries, in particular—pivotal for the formation of a coalition, with high initial fees. In order to achieve such a scenario, about ten to twenty of the largest greenhouse gas emitters must coordinate and agree that coalition formation and the refunding scheme fail if any of them defects.^22^

As full participation by all countries at once is unlikely, it is useful to resort to sequential procedures where a subset of countries makes a start and the others follow later (see Andreoni 1998; Varian 1994). We envision four steps. First, as suggested in the last paragraph, a set of large countries could initiate the system by paying initial fees and form a Refunding Club. In particular, if the US, the EU countries, China and maybe India would start the system with significant initial fees, this would constitute the Refunding Club in which the largest share of greenhouse gases are emitted. In addition, if wealthy countries pay substantially larger initial fees than the modesty approach suggests, such a Refunding Club would be powerful enough to slow down climate change significantly.

Second, smaller rich countries could follow, which would increase the initial wealth. In the third and fourth steps, larger and smaller developing countries could be invited to join the Refunding Club. Regarding the payment of initial fees, they should be treated differently, as we will discuss next.

The successful implementation of a refunding scheme only depends on raising the minimum initial global fund, but not on the individual countries’ contributions to it. Thus, within a coalition, the refunding scheme is able to disentangle efficiency from distributional concerns. Yet, in reality, the distribution of initial fees to raise the initial global fund is of great importance. For example, many developing countries may lack the necessary wealth to pay the initial fees, or countries in transition may refuse to pay high initial fees by arguing that historically, the current atmospheric greenhouse gas concentrations were caused by industrialized countries. To induce participation, payment of initial fees could be differentiated according to different distributional criteria such as stage of development, current greenhouse emissions or historic responsibility with respect to atmospheric greenhouse gas concentrations.^23^ Thus, refunding schemes and the payment of initial fees can be made compatible with the concept of “common but differentiated responsibilities and respective capabilities”, as detailed in Article 4 of the Paris Agreement (UNFCCC 2015). To sum up, allocating the burden of the initial fees is a tool that can solve distributional concerns, since they indirectly implement transfers across countries.

### Raising Initial Fees

Even differentiated initial fees cannot circumvent the problem that the sustainable refunding scheme relies on successfully raising the minimum initial global fund. As this fund may be quite large, even in the modesty solution, we outline two ways in which it might be financed.

Raising the minimum initial fund in full at the beginning of the treaty is not necessary. We can also achieve a coalition solution in which a smaller amount of money of money is paid repeatedly. To see this, let $\{R_t\}_{t=0}^T$ be the sequence of refunds in a solution envisioned by a coalition. In addition, we define the sequence of fees $f_t(\Delta )$ for a time span $\Delta > 0$ by

18 $$\begin{aligned} f_t(\Delta ) = \sum \limits _{\tau = 1}^{\Delta } \frac{R_{t+\tau }}{(1+\rho )^\tau }\ . \end{aligned}$$

If $f_t(\Delta )$ is paid into the fund at times $t=0,\Delta , 2\Delta ,\ldots$, the net present value of the fund is equal to the initial fund $f_0= \sum _{t=1}^{T-1}\left[ \frac{R_t}{(1+\rho )^t}\right]$ and, thus, the same solution can be achieved as when $f_0$ is initially paid in full.

With the repeated payments scheme, we face a trade-off between high initial fees and the property of an RS that transforms an intertemporal climate-policy problem into a static public-goods problem. In particular, if the time span $\Delta$ is short, the solution of the problem of a coalition relies on the repeated commitment of all countries, as the initial participation problem would have to be solved whenever new payments have to be made. Therefore $\Delta$ should not be too small.

If the repeated solution to the initial participation problem turns out to be a major obstacle to international cooperation, raising the initial fees by allowing countries to borrow money may be more advisable. Countries could then borrow either from the international capital market or directly from the administering agency of the RS. In the latter case, no actual initial money flows would be needed, since the initial fee would then simply be a liability of countries at the administration agency. In turn, future refund claims would be reduced or could even become negative, as countries would have to pay interest and ultimately pay back their liabilities to the agency. Hence, borrowing from the agency appears like a Munchhausen solution to the problem of raising initial fees.

However, at least two problems may arise. First, if countries borrow a large amount from the agency, they may later only receive a small payment or may even have to pay when refunds and repayment obligations are netted. Hence, countries might be tempted to renounce high abatement efforts and to default on their repayment obligations to the agency. The country would then lose all claims to refunds. However, as such refunds are small when abatement efforts are small, such a strategy may be profitable. That is, a country could choose to default against the administering agency and could free-ride on the abatement efforts of other countries even if it has signed the treaty and has borrowed from the agency. Such considerations suggest that countries should rather be made to borrow on the international capital market.

Second, if countries borrow a large amount on international capital markets, the default risk may rise if outstanding government debt is already at a high level. If the country needs to pay a higher interest rate than the risk-free rate, as investors demand a positive risk premium, further borrowing may increase the default risk, as refunds are insufficient to cover interest-rate payments. In such cases, it is more efficient if part of the initial fund is being raised by taxes over several periods.

### Information Requirements and Reaction to Unforeseen Shocks

The design of any RS rests on the bold assumption that all exogenous parameters are constant and, in particular, that they are known ex ante. These are demanding informational requirements.

We distinguish between temporary and permanent changes of parameters. Temporary shocks to the parameters do not inhibit the long-run behavior of the refunding scheme, because of the global convergence to the first-best solution. It is likely that initial expectations about the discount/interest rate $\delta$, the abatement cost parameters $\alpha _i$, the damage cost parameters $\beta _i$, and the business-as-usual emissions $\epsilon _i$ turn out to be incorrect and that at some time *t*, new information on one or several of these parameter arrives. In particular, technological progress may substantially change the abatement cost parameters.

Permanent changes in the exogenously given parameters would, in general, change the necessary refunds for a RS corresponding to a given feasible coalition abatement path.^24^ Moreover, in general, also the aspired coalition abatement path itself would change, for example, the abatement path and the degree of modesty that renders the grand coalition stable.

To accommodate permanent changes in the exogenous parameters, the RS could include a clause that the values of these parameters are re-evaluated on a regular basis (e.g., every ten years) and that the fund’s wealth is corrected accordingly, either by raising additional money from the members or paying back wealth to member countries.

Even if revision cannot be done frequently, the refunding scheme offers some built-in corrections. For example, when marginal damages increase, also the individually optimal abatement efforts for a given refunding scheme increase. However, the extent to which such built-in reactions to parameter changes correct deviations from the first-best solution or from a coalition solution is beyond the scope of this paper, but constitutes an important avenue for future research.

### Sustainable Climate Treaties in Overlapping Generation Frameworks

So far, we have focused on the properties of a RS and on how the implementation of such a scheme can be eased through repeated payments or through the use of capital markets. Still, we have assumed so far that the countries’ interest can be represented by a long-lived social planner.

The implementation of sustainable refunding schemes is more difficult in overlapping generation models, in which each generation is predominantly concerned about its own welfare. Then, setting up a refunding scheme hurts the old (existing) generations and benefits future generations—and possibly young existing generations—via two channels. First, the benefits from higher abatement today mainly accrue to future generations. This was the focus of important papers by Bovenberg and Heijdra (1998, 2002).^25^ Public debt policies can help redistribute the welfare gains from increased abatement more equally across generations. Essentially, by issuing (more) public debt today and by having future generations pay it back, the welfare of current generations can be increased at the expense of future generations. Additional effects such as a potential crowding out of physical capital investments and the reduction of distortionary taxation affect the balance between current and future generations.

Second, current generations must set up the fund and thus are, in principle, required to channel some of their savings towards the payment of initial fees. Since the global fund also invests, such savings may not necessarily decrease capital accumulation, but as future generations inherit the global fund for their own refunding, setting up the global fund decreases the welfare of current generations. Again, to redistribute the burden of setting up the global fund more equally across generations, one might implement repeated payments, as discussed above, or again, public debt can be used to increase the disposable income of current old generations.

In principle, the use of public debt can engineer trade among generations, can ease the implementation of sustainable refunding schemes and opens up the possibilities to achieve Pareto-improving climate policies across generations. However, with much higher public debt levels after the Covid-19 pandemic in many countries, the scope for further increases of public debt is quite limited.

## Conclusion

In this paper, we have shown that a refunding scheme, which is a rule-based treaty offering monetary incentives for emission abatement to member countries that are proportional to their relative abatement efforts, may promote sustained international cooperation with respect to anthropogenic climate change. The RS provides a simple blueprint for an international treaty on climate change and depends on a small number of parameters.

Yet, the RS is no panacea, as free-rider problems have no perfect solutions. For example, our numerical illustration shows that implementing a stable grand coalition in the modesty approach, which stabilizes average surface temperature at approximately 2.4 $^{\circ }\hbox {C}$, requires funds in the amount of 2.64 tril. USD. Given that the Green Climate Fund (GCF),^26^ the existing real world institution closest to our refunding scheme, has set itself the goal to raise 100 bil. USD per year starting from 2020, but has great difficulties in securing the pledges for these sums, such a sum seems considerably high. Yet, it is comparable to the sums raised to counter other global crises such as the latest financial crisis or the Corona pandemic. Still, the industrialized countries would have to shoulder a large share of the initial fees.

We stress that a decisive difference between the GCF and the RS is that the RS refunds money according to a simple and transparent rule (which is already known when initial fees are raised), while the GCF is governed by a 24-member board who decides which projects will be financed by the fund *after* the money has been raised.

No doubt, the practical implementation of the refunding schemes in a Refunding Club developed in this paper requires a variety of additional considerations. In the last section, we have discussed how to achieve better initial participation, and we have outlined several ways of raising initial fees. Other issues, such as the governance of the administering agency, or the stimulation of technological progress in abatement technologies will need thorough investigation in future research.

## Appendix

### Proof of Proposition 1

The decentralized solution is a special case of the second stage of the modest coalition formation game, as detailed in Sect. 5. Thus, Proposition 6, which states that there exists a unique subgame perfect Nash equilibrium of the second stage of the game for any given membership structure $\mathcal {C}$ and modesty parameter $\mu$ also covers the decentralized solution. In fact, the decentralized solution is characterized by $\mathcal {C}=\varnothing$, i.e., the coalition is an empty set and all countries $i\in \mathcal {I}$ do not participate in the treaty.

In the solution () of the proof of Proposition 6 the decentralized solution corresponds to $x=0$ and $y=\Gamma$ implying also $\bar{A}^\mathcal {C}=0$, $\bar{A}^\mathcal {NC}=\mathcal {E}$ and $\bar{s} = \frac{1-\delta }{\delta }\mathcal {E}$. Thus, we obtain for the aggregate emission abatement level $A_t = \sum _{i \in \mathcal {I}} a_t^i$ and the stock of aggregate cumulative emissions $s_t$:

19a $$\begin{aligned} A_{t}&= \mathcal {E} + B_2(T) (1-\lambda _2) \lambda _2^t - B_3(T) (1-\lambda _3) \lambda _3^t, \end{aligned}$$

19b $$\begin{aligned} s_t&= \bar{s} + B_2(t) \lambda _2^t + B_3(T) \lambda _3^t, \end{aligned}$$

with

20a $$\begin{aligned} \lambda _2&= \frac{1+\delta (1+\Gamma ) - \sqrt{[1+\delta (1+\Gamma )]^2-4\delta }}{2\delta }, \end{aligned}$$

20b $$\begin{aligned} \lambda _3&= \frac{1+\delta (1+\Gamma ) + \sqrt{[1+\delta (1+\Gamma )]^2-4\delta }}{2\delta }, \end{aligned}$$

and

21a $$\begin{aligned} B_2(T)&= - \frac{\mathcal {E} + (s_0-\bar{s})(1-\lambda _3)\lambda _3^{T}}{(1-\lambda _2) \lambda _2^T - (1-\lambda _3) \lambda _3^T}, \end{aligned}$$

21b $$\begin{aligned} B_3(T)&= \frac{\mathcal {E} + (s_0-\bar{s})(1-\lambda _2)\lambda _2^{T}}{(1-\lambda _2) \lambda _2^T - (1-\lambda _3) \lambda _3^T}\ . \end{aligned}$$

The individual countries’ abatement levels in the subgame perfect Nash equilibrium of the decentralized solution are given by:

22 $$\begin{aligned} a_t^i = \frac{\gamma _i}{\Gamma } A_{t}, \qquad \forall \ i\in \mathcal {I}, \quad t=0,\dots ,T\ . \end{aligned}$$

$$\square$$

### Proof of Proposition 2

Also the global social optimum is a special case of the second stage of the modest coalition formation game, as detailed in Sect. 5. Thus, Proposition 6, which states that there exists a unique subgame perfect Nash equilibrium of the second stage of the game for any given membership structure $\mathcal {C}$ and modesty parameter $\mu$ also covers the global social optimum. In fact, the global social optimum is characterized by $\mu =1$ and $\mathcal {C}=\mathcal {I}$, i.e., the coalition is the grand coalition encompassing all countries $i\in \mathcal {I}$ and fully internalizes all damages imposed by GHG emissions on all other countries.

In the solution () of the proof of Proposition 6 the global social optimum corresponds to $x=\mathcal {AB}$ and $y=0$ implying also $\bar{A}^\mathcal {C}=\mathcal {E}$, $\bar{A}^\mathcal {NC}=0$ and $\bar{S} = \frac{1-\delta }{\delta }\mathcal {E}$. Thus, we obtain for the aggregate emission abatement level $A_t = \sum _{i \in \mathcal {I}} a_t^i$ and the stock of aggregate cumulative emissions $s_t$:

23a $$\begin{aligned} A_{t}&= \mathcal {E} + B_2(T) (1-\lambda _2) \lambda _2^t - B_3(T) (1-\lambda _3) \lambda _3^t, \end{aligned}$$

23b $$\begin{aligned} s_t&= \bar{S} + B_2(t) \lambda _2^t + B_3(T) \lambda _3^t, \end{aligned}$$

with

24a $$\begin{aligned} \lambda _2&= \frac{1+\delta (1+\mathcal {AB}) - \sqrt{[1+\delta (1+\mathcal {AB})]^2-4\delta }}{2\delta }, \end{aligned}$$

24b $$\begin{aligned} \lambda _3&= \frac{1+\delta (1+\mathcal {AB}) + \sqrt{[1+\delta (1+\mathcal {AB})]^2-4\delta }}{2\delta }, \end{aligned}$$

and

25a $$\begin{aligned} B_2(T)&= - \frac{\mathcal {E} + (s_0-\bar{s})(1-\lambda _3)\lambda _3^{T}}{(1-\lambda _2) \lambda _2^T - (1-\lambda _3) \lambda _3^T}, \end{aligned}$$

25b $$\begin{aligned} B_3(T)&= \frac{\mathcal {E} + (s_0-\bar{s})(1-\lambda _2)\lambda _2^{T}}{(1-\lambda _2) \lambda _2^T - (1-\lambda _3) \lambda _3^T}\ . \end{aligned}$$

The individual countries’ abatement levels in the global social optimum are given by:

26a $$\begin{aligned} a_T^i&= 0, \qquad \forall \ i\in \mathcal {I}, \end{aligned}$$

26b $$\begin{aligned} a_t^i&= \frac{A_{t}}{\alpha _i\mathcal {A}}, \qquad \forall \ i\in \mathcal {I}, \quad t=0,\dots ,T-1\ . \end{aligned}$$

$$\square$$

### Proof of Proposition 3

The situation, in which a set of non-member countries strategically choose emission abatement levels such as to minimize their own domestic costs is similar to the second stage of the coalition formation game, as discussed in Sect. 5 and Proposition 6. The only difference is that the coalition $\mathcal {C}$ is following an exogenously given emission abatement paths instead of strategically reacting to the emission abatement choices of all non-member countries $i \notin \mathcal {C}$. Thus, existence and uniqueness of the subgame perfect equilibrium can be shown perfectly analogously to the proof of Proposition 6 by assuming an exogenously given aggregate emission abatement path $A^{\mathcal {C}}_t$ of the coalition.

Thus, we directly obtain the following system of first-order linear difference equations for the aggregated emission abatement levels of non-member countries $A^{\mathcal {NC}}_t = \sum _{i \notin \mathcal {C}} a_t^i$ and the stock of aggregated cumulative emissions $s_t$ for some exogenously given path of aggregate emission abatement $A^\mathcal {C}_t$ of the coalition $\mathcal {C}$:

27a $$\begin{aligned} A_{t+1}^\mathcal {NC}&= \left( \frac{1}{\delta }+\Gamma ^\mathcal {NC} \right) A_{t}^\mathcal {NC} -\Gamma ^\mathcal {NC} s_t - \Gamma ^\mathcal {NC}\left( \mathcal {E} - A_t^\mathcal {C}\right) , \end{aligned}$$

27b $$\begin{aligned} s_{t+1}&= -A_t^\mathcal {NC} + s_t +\mathcal {E} - A_t^\mathcal {C}\ . \end{aligned}$$

Introducing the matrix *M*:

28 $$\begin{aligned} M = \begin{pmatrix} \frac{1}{\delta } + \Gamma ^\mathcal {NC} &{} -\Gamma ^\mathcal {NC}\\ -1 &{} +1 \end{pmatrix}, \end{aligned}$$

we rewrite the system () in matrix form:

29 $$\begin{aligned} \begin{pmatrix} A_{t+1}^\mathcal {NC}\\ s_{t+1} \end{pmatrix} = M \cdot \begin{pmatrix} A_{t}^\mathcal {NC}\\ s_{t} \end{pmatrix} + \begin{pmatrix} - \Gamma ^\mathcal {NC} \left( \mathcal {E}-A^\mathcal {C}_t\right) \\ \mathcal {E}-A^\mathcal {C}_t \end{pmatrix}\ . \end{aligned}$$

The general solution of the matrix equation (29) is given by:

30 $$\begin{aligned} \begin{pmatrix} A_{t}^\mathcal {NC}\\ s_{t} \end{pmatrix} = \begin{pmatrix} \bar{A}^\mathcal {NC}_t\\ \bar{s}_t \end{pmatrix} + B_1(T) \nu _1 \lambda _1^t + B_2(T) \nu _2 \lambda _2^t, \end{aligned}$$

where $\bar{A}^\mathcal {NC}_t$ and $\bar{s}_t$ denote particular solutions to (29),^27^$\lambda _i$ are the eigenvalues and $\nu _i$ the eigenvectors of the matrix *M*, and $B_i(T)$ are constants determined by the initial and terminal conditions of the stock and the emission abatement levels ($i=1,2$).

The particular solutions are given by:

31 $$\begin{aligned} \begin{pmatrix} \bar{A}^\mathcal {NC}_t\\ \bar{s}_t \end{pmatrix} = \sum _{t'=0}^{t-1} M^{t'} \cdot \begin{pmatrix} - \Gamma ^\mathcal {NC} \left( \mathcal {E}-A^\mathcal {C}_{t'}\right) \\ \mathcal {E}-A^\mathcal {C}_{t'} \end{pmatrix}\ . \end{aligned}$$

In addition, for the matrix *M* we derive the following eigenvalues $\lambda _i$ ($i=1,2$):

32a $$\begin{aligned} \lambda _1&= \frac{1+\delta \left( 1+\Gamma ^\mathcal {NC}\right) -\sqrt{\left[ 1+\delta \left( 1+\Gamma ^\mathcal {NC}\right) \right] ^2-4\delta }}{2\delta }, \end{aligned}$$

32b $$\begin{aligned} \lambda _2&= \frac{1+\delta \left( 1+\Gamma ^\mathcal {NC}\right) +\sqrt{\left[ 1+\delta \left( 1+\Gamma ^\mathcal {NC}\right) \right] ^2-4\delta }}{2\delta }, \end{aligned}$$

and eigenvectors ($$i=1,2$$):

33a $$\begin{aligned} \nu _1&= \left\{ 1-\lambda _1, 1 \right\} , \end{aligned}$$

33b $$\begin{aligned} \nu _2&= \left\{ 1-\lambda _2, 1 \right\} \ . \end{aligned}$$

Inserting into Eq. (30) yields:

34a $$\begin{aligned} A_{t}^\mathcal {NC}&= \bar{A}^\mathcal {NC}_t + B_1(T)(1-\lambda _1)\lambda _1^t + B_2(T)(1-\lambda _2)\lambda _2^t, \end{aligned}$$

34b $$\begin{aligned} s_{t}&= \bar{s}_t + B_1(T) \lambda _1^t + B_2(T) \lambda _2^t \end{aligned}$$

The constants $B_i(T)$ ($i=1,2$) are derived from the initial stock $s_0$ and the terminal condition $A_{T}^\mathcal {NC} = 0$, which implies

35a $$\begin{aligned} B_1(T)&= - \frac{\bar{A}^\mathcal {NC}_T + (s_0-\bar{s}_0)(1-\lambda _2)\lambda _2^{T}}{(1-\lambda _1) \lambda _1^T - (1-\lambda _2) \lambda _2^T}, \end{aligned}$$

35b $$\begin{aligned} B_2(T)&= \frac{\bar{A}^\mathcal {NC}_T + (s_0-\bar{s}_0)(1-\lambda _1)\lambda _1^{T}}{(1-\lambda _1) \lambda _1^T - (1-\lambda _2) \lambda _2^T}\ . \end{aligned}$$

The individual countries’ abatement levels in the subgame perfect Nash equilibrium are given by:

36a $$\begin{aligned} a_T^i&= 0, \qquad \forall \ i \in \mathcal {I}, \end{aligned}$$

36b $$\begin{aligned} a_t^i&= \frac{\gamma _i}{\Gamma ^\mathcal {NC}} A^\mathcal {NC}_{t}, \qquad \forall \ i\notin C, \quad t=0,\dots ,T-1\ . \end{aligned}$$

$$\square$$

### Proof of Proposition 4

First, note that if the RS is able to incentivize all member countries $i \in \mathcal {C}$ to implement the aspired abatement paths $\left\{ \tilde{a}_t^i\right\} _{t=0,\dots ,T}^{i \in \mathcal {C}}$ we can use Proposition 3 to determine the emission abatement paths $\left\{ \check{a}_t^i\right\} _{t=0,\dots ,T}^{i \notin \mathcal {C}}$ for all non-member countries $i \notin \mathcal {C}$ in the subgame perfect Nash equilibrium. Thus, it suffices to show that given these emission abatement paths of non-member countries $\left\{ \check{a}_t^i\right\} _{t=0,\dots ,T}^{i \notin \mathcal {C}}$, there exists a RS that implements the aspired abatement paths $\left\{ \tilde{a}_t^i\right\} _{t=0,\dots ,T}^{i \in \mathcal {C}}$ for all coalition members $i \in \mathcal {C}$ as a subgame perfect Nash equilibrium. For further use, we define the aggregated emission abatement level of all non-member countries $i \notin \mathcal {C}$ in period *t* in the subgame perfect Nash equilibrium by $\check{A}_t^\mathcal {NC} = \sum _{i \notin \mathcal {C}} \check{a}_t^i$.

To prove this, we assume that a set of countries $\mathcal {C}$ has joined a feasible RS characterized by a weighting scheme $\left\{ \lambda _t^i\right\} _{t=0,\dots ,T-1}^{i \in \mathcal {C}}$ and a sequence of refunds $\{R_t\}_{t=0,\dots ,T-1}$ by paying an initial fee $f_0^i$. We shall analyze the subgame perfect Nash equilibria of the RS by backward induction. In every step of the backward induction, we show that

1. the objective function of each country *i* is strictly concave,
2. there exists a feasible weighting scheme $\{\tilde{\lambda }_t^i\}^{i \in \mathcal {C}}$ and a feasible refund $\tilde{R}_t$ such that the aspired abatement levels $\{\tilde{a}_t^i\}^{i \in \mathcal {C}}$ are consistent with the necessary and sufficient conditions of the subgame perfect Nash equilibrium of the subgame starting in period *t* and
3. the aspired abatement levels $\{\tilde{a}_t^i\}^{i \in \mathcal {C}}$ are the unique solution solving the necessary and sufficient conditions of subgame perfection of the subgame starting in period *t*,

given the aspired abatement levels $\{\tilde{a}_{t+1}^i\}^{i \in \mathcal {C}}$ constitute the unique subgame perfect Nash equilibrium outcome of the subgame starting in period $t+1$.

Assuming that there exists a unique subgame perfect equilibrium for the subgame starting in period $t+1$ with a stock of cumulative greenhouse gas emissions $s_{t+1}$, for all countries $i \in \mathcal {C}$, we denote country *i*’s equilibrium payoff for this subgame by $W^i_{t+1}(s_{t+1})$. Then country *i*’s best response in period *t*, $\bar{a}_t^i$, is determined by the solution of the optimization problem

37 $$\begin{aligned} V_t^i(s_t)|A_t^{-i} = \max \limits _{a_t^i} \left\{ \delta W_{t+1}^i(s_{t+1}) -\frac{\alpha _i}{2}(a_t^i)^2 - \frac{\beta _i}{2}s_t^2 + r^i_t\right\} , \end{aligned}$$

subject to Eq. (3), $W_{T+1}^i(s_{T+1})\equiv 0$, and given the sum of the abatement efforts of all other countries $A_t^{-i} = \sum _{j\ne i}a_t^j$. Differentiating Eq. (37) with respect to $a^i_t$ and setting it equal to zero yields

38 $$\begin{aligned} \alpha _i \bar{a}_t^i = -\delta {W_{t+1}^i}' (\bar{s}_{t+1})+\left. \frac{\partial r^i_t}{\partial a^i_t}\right| _{a^i_t=\bar{a}^i_t}\ \end{aligned}$$

where $$\bar{s}_{t+1} = s_t+\mathcal {E} - \bar{a}_t^i - A_t^{-i}$$ and

39 $$\begin{aligned} \frac{\partial r^i_t}{\partial a^i_t} = {\left\{ \begin{array}{ll} \lambda _t^i R_t \displaystyle \frac{A^{\mathcal {C}-i}_t}{(a^i_t+A^{\mathcal {C}-i}_t)^2}\ , \quad &{}t=1,\dots ,T-1,\\ 0, \quad &{}t=T\ . \end{array}\right. } \end{aligned}$$

Differentiating w.r.t. $s_t$ and applying the envelope theorem yields

40 $$\begin{aligned} -{V_t^i}'(s_t)|A_t^{-i} = \beta _i s_t-\delta {W_{t+1}^i}'(s_{t+1})\ . \end{aligned}$$

Starting with period $t=T$, we first note that the maximization problem of all countries is strictly concave, as $W_{T+1}(s_{T+1})\equiv 0$ and $r_T = f_T/|\mathcal {C}|$. Thus, Eq. (38) characterizes the best response for all countries $i \in \mathcal {C}$, which is given by $\bar{a}_{T}^i = 0$ independently of the abatement choices of all other countries. As a consequence ${\hat{a}_T^i}=0$ for all $i \in \mathcal {C}$ is the subgame perfect Nash equilibrium of the game starting in period *T* and is also the aspired abatement level in period *T*, as $\tilde{a}^i_T=0$ for all $i \in \mathcal {C}$ for all feasible coalition abatement paths. Then, the equilibrium pay-off is given by $W^i_T(s_T)=V^i_T(s_T)|\hat{A}^{-i}_T$, which is strictly concave:

41 $$\begin{aligned} W^i_T(s_T) = -\frac{\beta _i}{2}s_T^2 + \frac{f_T}{|\mathcal {C}|} \qquad \Rightarrow \qquad W_{T}''(s_T) = -\beta _i\ . \end{aligned}$$

Now, we analyze the subgame starting in period *t* assuming that their exists a weighting scheme $\{\tilde{\lambda }_{t'}^i\}_{{t'}=t,\dots ,T-1}^{i \in \mathcal {C}}$ and a sequence of refunds $\left\{ \tilde{R}_{t'}\right\} _{{t'}=t,\dots ,T-1}$ such that the outcome of the unique subgame perfect Nash equilibrium of the subgame starting in period $t+1$ coincides with the aspired coalition abatement paths $\left\{ \tilde{a}_{t'}^i\right\} _{{t'}=t+1,\dots ,T}^{i \in \mathcal {C}}$. In addition, we assume that $W_{t+1}^i(s_{t+1})$ is strictly concave. Then, also the optimization problem of country $i \in \mathcal {C}$ in period *t* is strictly concave

42 $$\begin{aligned} \delta {W_{t+1}^i}''(s_{t+1}) - \alpha _i + \frac{\partial ^2 r^i_t}{(\partial a^i_t)^2}< 0\ . \end{aligned}$$

As a consequence, there exists a unique best response $\bar{a}^i_t$ for all countries $i \in \mathcal {C}$ given the emission abatements of all other countries $j \ne i$, which is given implicitly by (38):

43 $$\begin{aligned} \alpha _i \bar{a}^i_t - \lambda _t^i R_t \frac{A^{\mathcal {C}-i}_t}{(\bar{a}^i_t+A^{\mathcal {C}-i}_t)^2} = -\delta {W_{t+1}^i}' (\bar{s}_{t+1})\ . \end{aligned}$$

As, by assumption, $-{W^i_{t'}}'(s_{t'})=-{V^i_{t'}}'(s_{t'})|\hat{A}_{t'}^{-i}$ for all $t'\ge t+1$ we can exploit Eq. (40) to obtain the following Euler equation:

44 $$\begin{aligned} \alpha _i \bar{a}^i_t - \lambda _{t}^i R_t \frac{A^{\mathcal {C}-i}_t}{(\bar{a}^i_t+A^{\mathcal {C}-i}_t)^2} = \delta \beta _i \bar{s}_{t+1} + \delta \alpha _i \tilde{a}_{t+i}^i -\delta \tilde{\lambda }_{t+1}^i \tilde{R}_{t+1} \frac{\tilde{A}_{t+1}^{\mathcal {C}-i}}{(\tilde{A}^\mathcal {C}_{t+1})^2}\ . \end{aligned}$$

Inserting $$\bar{s}_{t+1} = s_t + \mathcal {E}-\bar{a}_t^i - A_{t}^{\mathcal {C}-i}-\check{A}_t^{\mathcal {NC}}$$ yields:

45 $$\begin{aligned} \alpha _i \bar{a}^i_t + \delta \beta _i (\bar{a}_t^i + A_{t}^{\mathcal {C}-i}) - \lambda _t^i R_t \frac{A^{\mathcal {C}-i}_t}{(\bar{a}^i_t+A^{\mathcal {C}-i}_t)^2} = \tilde{C}_t^i, \end{aligned}$$

with

46 $$\begin{aligned} \tilde{C}_t^i = \delta \beta _i \left( s_t+\mathcal {E}-\check{A}_t^{\mathcal {NC}}\right) + \delta \alpha _i \tilde{a}_{t+i}^i -\delta \tilde{\lambda }^i_{t+1} \tilde{R}_{t+1} \frac{\tilde{A}_{t+1}^{\mathcal {C}-i}}{\left( \tilde{A}^\mathcal {C}_{t+1}\right) ^2}\ . \end{aligned}$$

First, we show that there exist unique $\tilde{\lambda }_t^i$ and $\tilde{R}_t$ such that choosing the aspired coalition abatement level $\tilde{a}_t^i$ is an equilibrium strategy for all countries $i \in \mathcal {C}$. Inserting aspired abatement levels $\tilde{a}_t^i$ and rearranging Eq. (45), we obtain

47 $$\begin{aligned} \tilde{\lambda }_t^i \tilde{R}_t = \left( \tilde{A}^\mathcal {C}_t\right) ^2 \left( \alpha _i \frac{\tilde{a}_t^i}{\tilde{A}_t^{\mathcal {C}-i}} + \delta \beta _i \frac{\tilde{A}^\mathcal {C}_t}{\tilde{A}_t^{\mathcal {C}-i}} -\frac{\tilde{C}_t^i}{\tilde{A}_t^{\mathcal {C}-i}}\right) \ . \end{aligned}$$

Taking into account that the weighting scheme adds up to one, i.e., $\sum _{j \in \mathcal {C}} \tilde{\lambda }_t^j\frac{\tilde{a}_t^j}{\tilde{A}_t^\mathcal {C}} = 1$, yields

48a $$\begin{aligned} \tilde{R}_t&= \tilde{A}^\mathcal {C}_t \sum _{j \in \mathcal {C}} \left[ \tilde{a}_t^j\left( \alpha _j \frac{\tilde{a}_t^j}{\tilde{A}_t^{\mathcal {C}-j}} + \delta \beta _j \frac{\tilde{A}^\mathcal {C}_t}{\tilde{A}_t^{\mathcal {C}-j}} -\frac{\tilde{C}_t^j}{\tilde{A}_t^{\mathcal {C}-j}}\right) \right] \ , \end{aligned}$$

48b $$\begin{aligned} \tilde{\lambda }_t^i&= \frac{\tilde{A}_t^\mathcal {C}\left( \alpha _i \frac{\tilde{a}_t^i}{\tilde{A}_t^{\mathcal {C}-i}} + \delta \beta _i \frac{\tilde{A}^\mathcal {C}_t}{\tilde{A}_t^{\mathcal {C}-i}} -\frac{\tilde{C}_t^i}{\tilde{A}_t^{\mathcal {C}-i}}\right) }{\sum _{j \in \mathcal {C}} \left[ \tilde{a}_t^j\left( \alpha _j \frac{\tilde{a}_t^j}{\tilde{A}_t^{\mathcal {C}-j}} + \delta \beta _j \frac{\tilde{A}^\mathcal {C}_t}{\tilde{A}_t^{\mathcal {C}-j}} -\frac{\tilde{C}_t^j}{\tilde{A}_t^{\mathcal {C}-j}}\right) \right] }\ . \end{aligned}$$

We now show that the aspired coalition abatement levels $\tilde{a}_t^i$ are the unique solution to the Euler equations of all countries $i \in \mathcal {C}$ given the weighting scheme $\left\{ \tilde{\lambda }_t^i\right\} ^{i \in \mathcal {C}}$ and the refund $\tilde{R}_t$. To this end, we express equation (45) in terms of $a_t^i$ and $A^\mathcal {C}_t$ and solve for $a_t^i$:

49 $$\begin{aligned} a_t^i = A^\mathcal {C}_t \underbrace{\frac{\tilde{\lambda }_t^i \tilde{R}_t + \tilde{C}_t^i A_t^\mathcal {C} - \delta \beta _i \left( A_t^\mathcal {C}\right) ^2}{\tilde{\lambda }_t^i \tilde{R}_t + \alpha _i \left( A_t^\mathcal {C}\right) ^2}}_{\equiv h_t^i(A^\mathcal {C}_t)} = A^\mathcal {C}_t h_t^i(A^\mathcal {C}_t)\ . \end{aligned}$$

Summing-up over all countries $i \in \mathcal {C}$ yields

50 $$\begin{aligned} \sum _{i \in \mathcal {C}} h_t^i(A^\mathcal {C}_t) = 1, \end{aligned}$$

which has to hold for $A^\mathcal {C}_t=\tilde{A}_t^\mathcal {C}$ and is a necessary condition for a Nash equilibrium. Differentiating $h_t^i(A^\mathcal {C}_t)$ with respect to $A^\mathcal {C}_t$, we obtain:

51 $$\begin{aligned} {h_t^i}'(A^\mathcal {C}_t) = \frac{\tilde{\lambda }_t^i \tilde{R}_t \tilde{C}_t^i -2(\alpha _i+\delta \beta _i)\tilde{\lambda }_t^i \tilde{R}_t A^\mathcal {C}_t - \alpha _i \tilde{C}_t^i \left( A_t^\mathcal {C}\right) ^2}{\left[ \tilde{\lambda }_t^i \tilde{R}_t+ \alpha _i \left( A_t^\mathcal {C}\right) ^2\right] ^2}\ . \end{aligned}$$

Seeking the roots of ${h_t^i}'(A^\mathcal {C}_t)$ yields

52 $$\begin{aligned} {h_t^i}' (A^\mathcal {C}_t)= 0&\Leftrightarrow \underbrace{\tilde{\lambda }_t^i \tilde{R}_t \tilde{C}_t^i}_{\equiv x> 0} - \underbrace{2(\alpha _i+\delta \beta _i)\tilde{\lambda }_t^i \tilde{R}_t}_{\equiv y> 0} A^\mathcal {C}_t - \underbrace{\alpha _i \tilde{C}_t^i}_{\equiv z > 0} \left( A_t^\mathcal {C}\right) ^2 = 0, \end{aligned}$$

53 $$\begin{aligned}&\Leftrightarrow x - y A^\mathcal {C}_t -z \left( A^\mathcal {C}_t\right) ^2 = 0, \end{aligned}$$

54 $$\begin{aligned}&\Leftrightarrow A^\mathcal {C}_t = -\frac{y\pm \sqrt{y^2+4xz}}{2z}\ . \end{aligned}$$

Thus, for every $h_t^i(A^\mathcal {C}_t)$ there exist one positive collective abatement level $\bar{A}_t^i$ such that ${h_t^i}' (\bar{A}_t^i) = 0$. In addition it holds (taking into account Eq. (47)):

55a $$\begin{aligned} h_t^i (0)&= 1,&\quad h_t^i (\tilde{A}_t^\mathcal {C})&= \frac{\alpha _i \tilde{a}_t^i \tilde{A}^\mathcal {C}_t + \tilde{\lambda }_t^i \tilde{R}_t \left( 1-\frac{\tilde{A}_t^{\mathcal {C}-i}}{\tilde{A}_t^\mathcal {C}}\right) }{\tilde{\lambda }_t^i \tilde{R}_t + \alpha _i \left( \tilde{A}_t^\mathcal {C}\right) ^2} \in [0,1], \tilde{A}_t^\mathcal {C} \ne 0 \end{aligned}$$

55b $$\begin{aligned} {h_t^i}' (0)&= \tilde{C}_t^i > 0 ,&\quad {h_t^i}' (\tilde{A}_t^\mathcal {C})&< 0\ . \end{aligned}$$

Focusing attention to the positive half-space $A^\mathcal {C}_t \ge 0$, all $h_t^i(A^\mathcal {C}_t)$ start at 1 for $A^\mathcal {C}_t=0$. In addition, all $h_t^i(A^\mathcal {C}_t)$ exhibit a unique local extremum at $\bar{A}_t^i > 0$. As $h_t^{i'}(A^\mathcal {C}_t)$ is increasing at $A^\mathcal {C}_t=0$, the local extremum is a local maximum. This implies that all $h_t^i(A^\mathcal {C}_t)$ are increasing until $\bar{A}_t^i > 0$ and decreasing afterwards. This also implies that $\tilde{A}^\mathcal {C}_t > \bar{A}_t^i$ for all $i \in \mathcal {C}$, because $h^i_t(\tilde{A}_t^\mathcal {C}) < 1$, which can only happen for values $A^\mathcal {C}_t > \bar{A}_t^i$, as all $h_t^i(A^\mathcal {C}_t)$ start at 1 and further increase until the local extremum at $\bar{A}_t^i$. As $\tilde{A}_t^\mathcal {C} > \bar{A}_t^i$, this, in turn, implies that at $\tilde{A}_t^\mathcal {C}$, all $h_t^i(\tilde{A}_t^\mathcal {C}) \in [0,1]$ are monotonically decreasing.^28^ As a consequence, there exists no other value $A_t'$ such that $\sum _{i \in \mathcal {C}} h_t^i(A_t') = 1$. Then, only the aspired coalition abatement levels $\tilde{a}_t^i$ solve the Euler equations of all countries $i \in \mathcal {C}$ simultaneously for the weighting scheme $\tilde{\lambda }_t^i$ and the refund $\tilde{R}_t$.

Differentiating (40) with respect to $s_t$, we obtain

56 $$\begin{aligned} {V_t^i}''(s_t)|A_t^{-i} = \delta {W_{t+1}^i}''(\bar{s}_{t+1})-\beta _i\ . \end{aligned}$$

As ${W_t^i}(s_t) = {V_t^i}(s_t)|\hat{A}_t^{-i}$, this implies that the equilibrium pay-off $W^i_{t}(s_t)$ is strictly concave for all countries $i \in \mathcal {C}$.

Working backwards to $t=1$ yields a the unique subgame perfect Nash equilibrium outcome that is given by the aspired coalition abatement levels $\left\{ \tilde{a}_t^i\right\} _{t=0,\dots ,T}^{i \in \mathcal {C}}$, the abatement path $\left\{ \check{a}_t^i\right\} _{t=0,\dots ,T}^{i \notin \mathcal {C}}$ of all non-members countries $i \notin \mathcal {C}$ and the corresponding path of cumulative greenhouse gas emissions $\{s_t\}_{t=0,\dots ,T}$ .

It remains to show that the RS is feasible, i.e., the weighting scheme $\{\tilde{\lambda }_t^i\}_{i=1}^n$ and the refund $\tilde{R}_t$ are non-negative for all $t=0,\dots ,T-1$, for all feasible coalition abatement paths $\left\{ \tilde{a}_t^i\right\} _{t=0,\dots ,T}^{i \in \mathcal {C}}$. As ${W_t^i}(s_t) = {V_t^i}(s_t)|\hat{A}_t^{-i}$, we can consecutively apply Eq. (40), insert into Eq. (38) and evaluate in the subgame perfect Nash equilibrium:

57 $$\begin{aligned} \alpha _i \tilde{a}_t^i - \tilde{\lambda }_t^i \tilde{R}_t \frac{\tilde{A}_t^{\mathcal {C}-i}}{\left( \tilde{A}^\mathcal {C}_t\right) ^2} = \delta \beta _i \sum _{\tau =t+1}^{T} \delta ^{\tau -(t+1)} s_\tau \ ,\quad t=0,\dots ,T-1\ . \end{aligned}$$

The corresponding equation in the decentralized solution yields:

58 $$\begin{aligned} \alpha _i \hat{a}_t^i = \delta \beta _i \sum _{\tau =t+1}^{T} \delta ^{\tau -(t+1)} \hat{s}_\tau ,\quad t=0,\dots ,T-1\ . \end{aligned}$$

By construction $\tilde{a}_t^i > \hat{a}_t^i$ for all $i \in \mathcal {C}$ and $t=0,\dots ,T-1$. As a consequence, it also holds that $\hat{s}_t > s_t$ for all $t=0,\dots ,T$. This, in turn, implies that $\{\tilde{\lambda }_t^i\}_{t=0,\dots ,T-1}^{i \in \mathcal {C}} > 0$ and $\left\{ \tilde{R}_t\right\} _{t=0,\dots ,T-1} > 0$. $\square$

### Proof of Proposition 5

The first part of Proposition directly follows from Eq. (14).

To show that the any feasible RS can always be implemented as a Pareto improvement over the decentralized solution, we introduce the following abbreviation: Denote the net present value of the discounted sum of abatement costs and environmental damage costs of country *i* in the decentralized solution and the RS by $\hat{K}_i$ and $\tilde{K}_i$, respectively:

59a $$\begin{aligned} \hat{K}_i&= \sum _{t=0}^T \left[ \frac{\alpha _i}{2}\left( \hat{a}_t^i\right) ^2 + \frac{\beta _i}{2}\hat{s}_t^2\right] , \end{aligned}$$

59b $$\begin{aligned} \tilde{K}_i&= \sum _{t=0}^T \left[ \frac{\alpha _i}{2}\left( \tilde{a}_t^i\right) ^2 + \frac{\beta _i}{2} \tilde{s}_t^2\right] \ . \end{aligned}$$

In addition, let $\tilde{f}_0^i$ be the net present value of the discounted sum of refunds that country $i \in \mathcal {C}$ receives in the RS:

59c $$\begin{aligned} \tilde{f}_0^i = \sum _{t=1}^{T-1} \frac{\tilde{\lambda }_i \tilde{R}_t}{(1+\rho )^t} \left( \frac{a^i_t}{\sum _{j \in \mathcal {C}} a^j_t} \right) . \end{aligned}$$

By construction, all countries $i \in \mathcal {C}$ are better off in the RS than in the decentralized solution if their initial fees were equal to zero. The reason is that environmental damage costs are smaller under the refunding scheme and abatement costs minus refunds are smaller compared to the decentralized solution. Otherwise, it would not have been in the countries’ best interest to choose the aspired coalition abatement levels. Define the difference in terms of net present value between the RS and the decentralized solution by $\hat{f}_0^i$:

60 $$\begin{aligned} \hat{f}_0^i = \hat{K}_i - \tilde{K}_i + \tilde{f}_0^i > 0\ . \end{aligned}$$

Note that $\hat{f}_0^i$ is the initial fee that would leave country $i \in \mathcal {C}$ indifferent between the RS and the decentralized solution. Summing-up over all countries $i \in \mathcal {C}$, we obtain:

61 $$\begin{aligned} \sum _{i \in \mathcal {C}} \hat{f}_0^i = \sum _{i \in \mathcal {C}} \left[ \hat{K}_i - \tilde{K}_i + \tilde{f}_0^i\right] = \sum _{i \in \mathcal {C}} \left[ \hat{K}_i - \tilde{K}_i\right] + \tilde{f}_0 > \tilde{f}_0\ . \end{aligned}$$

Thus, it is always possible to find a set of initial fees $f_0^i$ such that $\sum _{i \in \mathcal {C}} f_0^i = \tilde{f}_0$ and, in addition, $f_0^i < \hat{f}_0^i$ for all $i \in \mathcal {C}$. $\square$

### Proof of Proposition 6

In line with the literature, we assume that in the second stage both the modesty parameter $\mu$ and the membership structure are given and common knowledge. Then, the coalition acts as one player in the non-cooperative game, in which the coalition and all other non-member countries choose emission abatement levels to maximize their objective. We assume that in each period $t=0,\dots ,T$ the previous emission abatement choices of all players are common knowledge before all players simultaneously decide on emission abatement levels in period *t*. The subgame perfect Nash equilibrium is derived by backward induction.

For a given modesty parameter $\mu$ and a given membership structure $\mathcal {C}$, the coalition is supposed to set emission abatement levels such as to solve optimization problem (15) subject to the equation of motion for aggregate cumulative emissions (3) and given the emission abatement levels of all non-member countries. To solve the problem recursively, we introduce the value function:

62 $$\begin{aligned} V_t^\mathcal {C}(s_t)|A_t^{-\mathcal {C}} = \max _{\{a_t^i\}_{i\in C}} \left\{ \delta W_{t+1}^C(s_{t+1}) - \sum _{i\in C}\left[ \frac{\alpha _i}{2}(a_t^i)^2+\mu \frac{\beta _i}{2}s_t^2\right] \right\} , \end{aligned}$$

where $A_t^{-\mathcal {C}}$ denotes the vector of emission abatement levels of all non-member countries, $V_t^\mathcal {C}(s_t)$ represents the negative of the total coalition costs accruing from period *t* onwards discounted to period *t* and $W_{t+1}^C(s_{t+1})$ is the coalition’s equilibrium pay-off of the subgame starting in period $t+1$ conditional on the stock of accumulated GHG gases $s_{t+1}$.

All non-member countries $i \notin \mathcal {C}$ seek to minimize the net present value of their own total domestic costs (6) subject to stock dynamics of cumulative global GHG emissions (3) and given the emission abatement levels of all other countries. Again, we introduce the value function:

63 $$\begin{aligned} V_t^i(s_t)|{A_t^{-i}} = \max _{\{a_t^i\}} \left\{ \delta W_{t+1}^i(s_{t+1}) - \left[ \frac{\alpha _i}{2}(a_t^i)^2+\frac{\beta _i}{2}s_t^2\right] \right\} ,\quad i\notin \mathcal {C}, \end{aligned}$$

where $A_t^{-i}$ denotes the vector of emission abatement levels of all other countries $j \ne i$, $V_t^i(s_t)$ represents the negative of the total country *i*’ costs accruing from period *t* onwards discounted to period *t* and $W_{t+1}^i(s_{t+1})$ is the country *i*’s equilibrium pay-off of the subgame starting in period $t+1$ conditional on the stock of accumulated GHG gases $s_{t+1}$.

Differentiating the value functions (62) and (63) with respect to $a^i_t$ and setting them equal to zero, we derive the following first-order conditions:

64a $$\begin{aligned} \alpha _i a^i_t&= -\delta {W^{\mathcal {C}}_{t+1}}'(s_{t+1}),\qquad \forall \ i \in \mathcal {C},\quad t=0,\dots ,T, \end{aligned}$$

64b $$\begin{aligned} \alpha _i a^i_t&= -\delta {W^{i}_{t+1}}'(s_{t+1}),\qquad \forall \ i \notin \mathcal {C},\quad t=0,\dots ,T\ . \end{aligned}$$

The optimization problems of the coalition and all non-member countries in period *t* are strictly concave if

65a $$\begin{aligned}&\delta {W_{t+1}^{\mathcal {C}}}''(s_{t+1}) - \alpha _i < 0,\qquad \forall \ i \in \mathcal {C},\quad t=0,\dots ,T, \end{aligned}$$

65b $$\begin{aligned}&\delta {W_{t+1}^{i}}''(s_{t+1}) - \alpha _i < 0,\qquad \forall \ i \notin \mathcal {C},\quad t=0,\dots ,T, \end{aligned}$$

in which case the first-order conditions () implicitly define the coalition’s and all non-member countries’ unique best response functions.

In addition, differentiating the value functions (62) and (63) with respect to $s_t$ and applying the envelope theorem yields

66a $$\begin{aligned} -{V_{t}^{\mathcal {C}}}'(s_t)|A_t^{-\mathcal {C}}&= \mu \mathcal {B}^\mathcal {C} s_t - \delta {W_{t+1}^{\mathcal {C}}}' (s_{t+1}),\qquad \forall \ i \in \mathcal {C},\quad t=0,\dots ,T, \end{aligned}$$

66b $$\begin{aligned} -{V_{t}^{i}}'(s_t)|A_t^{-i}&= \beta _i s_t - \delta {W_{t+1}^{i}}' (s_{t+1}),\qquad \forall \ i \notin \mathcal {C},\quad t=0,\dots ,T, \end{aligned}$$

where we have introduced the notation $\mathcal {B}^\mathcal {C} = \sum _{i\in \mathcal {C}} \beta _i$.

Starting from $W^\mathcal {C}_{T+1}(s_{T+1}) \equiv 0 \equiv W^i_{T+1}(s_{T+1})$ for all $i \in \mathcal {I}$, implying that the objective function of the optimization problem of the coalition and all non-member countries is strictly concave. As a consequence, Eq. () characterize the coalition’s and all non-member countries’ best response, which is given by $\bar{a}^i_T=0$ for all $I \in \mathcal {I}$ independently of the emission abatement choices of all other countries. As a consequence, $\tilde{a}^i_T=0$ for all $i \in \mathcal {C}$ and $\check{a}^i_T=0$ for all $i \notin \mathcal {C}$ is the unique and symmetric Nash equilibrium for the subgame starting in period *T* given the stock of cumulative greenhouse gas emissions $s_T$. The equilibrium pay-offs are given by $W^\mathcal {C}_T(s_T)= V^\mathcal {C}_T(s_T)|\hat{A}^{-\mathcal {C}}_T$ for the coalition and $W^i_T(s_T)=V^i_T(s_T)|\hat{A}^{-i}_T$ and are strictly concave:

67a $$\begin{aligned} W^\mathcal {C}_T(s_T)&= -\mu \frac{\mathcal {B}^\mathcal {C}}{2}s_T^2 \qquad \Rightarrow \qquad {W_{T}^\mathcal {C}}''(s_T) = -\mu \mathcal {B}^\mathcal {C}, \end{aligned}$$

67b $$\begin{aligned} W^i_T(s_T)&= -\frac{\beta _i}{2}s_T^2 \qquad \Rightarrow \qquad {W_{T}^i}''(s_T) = -\beta _i, \qquad \forall \ i \notin \mathcal {C}\ . \end{aligned}$$

As a consequence, the optimization problem of the coalition and all non-member countries is also strictly concave in period *T*.

Now assume there exists a unique subgame perfect Nash equilibrium for the subgame starting in period $t+1$ with a stock of greenhouse gas emissions of $s_{t+1}$ yielding equilibrium pay-offs $W^\mathcal {C}_{t+1}(s_{t+1})$ and $W^i_{t+1}(s_{t+1})$ to the coalition and all non-member countries $i \notin \mathcal {C}$, respectively, with ${W^\mathcal {C}_{t+1}}''(s_{t+1})<0$ and ${W^i_{t+1}}''(s_{t+1})<0$ . Then the optimization problem in period *t* is strictly concave for the coalition and all non-member countries $i \notin \mathcal {C}$, implying there exists a unique best response $\bar{a}^i_t$ for all countries $i \in \mathcal {I}$ given the emission abatements of all other countries $j \ne i$, which is given implicitly by

68a $$\begin{aligned} \alpha _i \bar{a}^i_t&= -\delta {W^{\mathcal {C}}_{t+1}}'(\bar{s}_{t+1}),\qquad \forall \ i \in \mathcal {C}, \end{aligned}$$

68b $$\begin{aligned} \alpha _i \bar{a}^i_t&= -\delta {W^{i}_{t+1}}'(\bar{s}_{t+1})\ ,\qquad \forall \ i \notin \mathcal {C}, \end{aligned}$$

where $\bar{s}_{t+1} = s_t+ \mathcal {E} - \bar{a}_t^i - A_t^{-i}$. As, by assumption, $-{W^\mathcal {C}_{t'}}'(s_{t'})=-{V^\mathcal {C}_{t'}}'(s_{t'})|\hat{A}_{t'}^{-\mathcal {C}}$ and $-{W^i_{t'}}'(s_{t'})=-{V^i_{t'}}'(s_{t'})|\hat{A}_{t'}^{-i}$ for all $t'\ge t+1$, we can exploit conditions () to obtain:

69a $$\begin{aligned} a_t^i&= \delta \tilde{a}^i_{t+1} + \mu \delta \frac{\mathcal {B}^{\mathcal {C}}}{\alpha _i} \left( s_t + \mathcal {E}- \sum _{j \in \mathcal {I}} a^j_t\right) ,\qquad \forall \ i \in \mathcal {C}, \end{aligned}$$

69b $$\begin{aligned} a_t^i&= \delta \check{a}^i_{t+1} + \delta \gamma _i \left( s_t + \mathcal {E}-\sum _{j \in \mathcal {I}} a^j_t\right) ,\qquad \forall \ i \notin \mathcal {C}\ . \end{aligned}$$

Summing up Eq. (69a) over all coalition members $i \in \mathcal {C}$ and Eq. (69b) over all non-member countries $i \notin \mathcal {C}$, we obtain the following equations for the aggregate abatement levels $A^\mathcal {C}_t = \sum _{i \in \mathcal {C}} a^i_t$ and $A^\mathcal {NC}_t = \sum _{i \notin \mathcal {C}} a^i_t$ of the coalition and all non-member countries, respectively:

70a $$\begin{aligned} A^\mathcal {C}_t&= \delta \tilde{A}^\mathcal {C}_{t+1} + \mu \delta \mathcal {A}^\mathcal {C} \mathcal {B}^{\mathcal {C}} \left( s_t + \mathcal {E}- A^\mathcal {C}_t - A^\mathcal {NC}_t\right) , \end{aligned}$$

70b $$\begin{aligned} A^\mathcal {NC}_t&= \delta \check{A}^\mathcal {NC}_{t+1} + \delta \Gamma ^\mathcal {NC}\left( s_t + \mathcal {E}- A^\mathcal {C}_t - A^\mathcal {NC}_t\right) , \end{aligned}$$

where we have used the abbreviation $\mathcal {A}^\mathcal {C} = \sum _{i \in \mathcal {C}} 1/\alpha _i$ and $\Gamma ^\mathcal {NC} = \sum _{i \notin \mathcal {C}} \gamma _i$. Solving this system of equations for $A^\mathcal {C}_t$ and $A^\mathcal {NC}_t$, we obtain the aggregate abatement levels of the coalition and non-member countries, respectively, for period *t* in the subgame perfect Nash equilibrium:

71a $$\begin{aligned} \tilde{A}^\mathcal {C}_t&= \frac{\delta \left[ \tilde{A}^\mathcal {C}_{t+1}\left( 1+\delta \Gamma ^{\mathcal {NC}}\right) + \mu \mathcal {A}^\mathcal {C} \mathcal {B}^{\mathcal {C}}\left( s_t + \mathcal {E}- \delta \check{A}^\mathcal {NC}_{t+1} \right) \right] }{1+\delta \Gamma ^{\mathcal {NC}}+\mu \delta \mathcal {A}^\mathcal {C} \mathcal {B}^{\mathcal {C}}}, \end{aligned}$$

71b $$\begin{aligned} \check{A}^\mathcal {NC}_t&= \frac{\delta \left[ \check{A}^\mathcal {NC}_{t+1}\left( 1+\mu \delta \mathcal {A}^\mathcal {C} \mathcal {B}^{\mathcal {C}}\right) + \Gamma ^{\mathcal {NC}} \left( s_t + \mathcal {E}- \delta \tilde{A}^\mathcal {C}_{t+1} \right) \right] }{1+\delta \Gamma ^{\mathcal {NC}}+\mu \delta \mathcal {A}^\mathcal {C} \mathcal {B}^{\mathcal {C}}}\ . \end{aligned}$$

Inserting $\tilde{A}^\mathcal {C}_t$ and $\check{A}^\mathcal {NC}_t$ back into Eq. () yields the unique equilibrium abatement level in period *t* for all countries $i \in \mathcal {I}$:

72a $$\begin{aligned} \tilde{a}_t^i&= \delta \tilde{a}^i_{t+1} + \mu \delta \frac{\mathcal {B}^{\mathcal {C}}}{\alpha _i} \left( s_t + \mathcal {E}- \tilde{A}^\mathcal {C}_t - \check{A}^\mathcal {NC}_t \right) ,\qquad \forall \ i \in \mathcal {C}, \end{aligned}$$

72b $$\begin{aligned} \check{a}_t^i&= \delta \check{a}^i_{t+1} + \delta \gamma _i \left( s_t + \mathcal {E}-\tilde{A}^\mathcal {C}_t - \check{A}^\mathcal {NC}_t \right) ,\qquad \forall \ i \notin \mathcal {C}\ . \end{aligned}$$

Differentiating () with respect to $s_t$, we obtain

73a $$\begin{aligned} {V_{t}^{\mathcal {C}}}''(s_t)|A_t^{-\mathcal {C}}&= \delta {W_{t+1}^{\mathcal {C}}}'' (s_{t+1}) -\mu \mathcal {B}^\mathcal {C},\qquad \forall \ i \in \mathcal {C}, \end{aligned}$$

73b $$\begin{aligned} {V_{t}^{i}}''(s_t)|A_t^{-i}&= \delta {W_{t+1}^{i}}''(s_{t+1}) - \beta _i,\qquad \forall \ i \notin \mathcal {C}\ . \end{aligned}$$

As ${W_t^\mathcal {C}}''(s_t) = {V_t^\mathcal {C}}''(s_t)|\hat{A}_t^{-\mathcal {C}}$ and ${W_t^i}''(s_t) = {V_t^i}''(s_t)|\hat{A}_t^{-i}$, this implies that the equilibrium pay-offs $W^\mathcal {C}_{t}(s_t)$ and $W^i_{t}(s_t)$ are strictly concave for the coalition and all non-member countries $i \notin \mathcal {C}$.

Working backwards until $t=0$ yields unique sequences of emission abatements $\{\tilde{a}^i_t\}^T_{t = 0}$ and $\{\tilde{a}^i_t\}^T_{t = 0}$ for all coalition countries $i\in \mathcal {C}$ and all non-member countries $i \notin \mathcal {C}$, respectively, and the corresponding sequence of the stock of cumulative greenhouse gas emissions $s_t$ ($t=0,\dots ,T$) that constitute the unique subgame perfect Nash equilibrium outcome of the second stage of the modest international environmental agreement.

Having established existence and uniqueness of the subgame perfect Nash equilibrium, we now employ Eq. () together with the equation of motion for the stock of aggregated cumulative emissions (3) to derive the following system of first-order linear difference equations:

74a $$\begin{aligned} A_{t+1}^\mathcal {C}&= \left( \frac{1}{\delta } + \mu \mathcal {A}^\mathcal {C} \mathcal {B}^\mathcal {C} \right) A_t^\mathcal {C} + \mu \mathcal {A}^\mathcal {C} \mathcal {B}^\mathcal {C} A_t^\mathcal {NC} - \mu \mathcal {A}^\mathcal {C} \mathcal {B}^\mathcal {C} s_t - \mu \mathcal {A}^\mathcal {C} \mathcal {B}^\mathcal {C} \mathcal {E}, \end{aligned}$$

74b $$\begin{aligned} A_{t+1}^\mathcal {NC}&= \Gamma ^\mathcal {NC} A_t^\mathcal {C} + \left( \frac{1}{\delta }+\Gamma ^\mathcal {NC} \right) A_{t}^\mathcal {NC} -\Gamma ^\mathcal {NC} s_t - \Gamma ^\mathcal {NC} \mathcal {E}, \end{aligned}$$

74c $$\begin{aligned} s_{t+1}&= - A_t^\mathcal {C} -A_t^\mathcal {NC} + s_t +\mathcal {E}\ . \end{aligned}$$

By introducing the abbreviations $x = \mu \mathcal {A}^\mathcal {C} \mathcal {B}^\mathcal {C}$ and $y = \Gamma ^\mathcal {NC}$ and the matrix *M*

75 $$\begin{aligned} M = \begin{pmatrix} \frac{1}{\delta } + x &{} x &{} -x\\ y &{} \frac{1}{\delta }+y &{} -y \\ -1 &{} -1 &{} +1 \end{pmatrix}, \end{aligned}$$

we rewrite the system () in matrix form:

76 $$\begin{aligned} \begin{pmatrix} A_{t+1}^\mathcal {C}\\ A_{t+1}^\mathcal {NC}\\ s_{t+1} \end{pmatrix} = M \cdot \begin{pmatrix} A_{t}^\mathcal {C}\\ A_{t}^\mathcal {NC}\\ s_{t} \end{pmatrix} + \begin{pmatrix} - x \mathcal {E}\\ - y \mathcal {E}\\ \mathcal {E} \end{pmatrix}\ . \end{aligned}$$

The general solution of the matrix equation (76) is given by:

77 $$\begin{aligned} \begin{pmatrix} A_{t}^\mathcal {C}\\ A_{t}^\mathcal {NC}\\ s_{t} \end{pmatrix} = \begin{pmatrix} \bar{A}^\mathcal {C}\\ \bar{A}^\mathcal {NC}\\ \bar{s} \end{pmatrix} + B_1(T) \nu _1 \lambda _1^t + B_2(T) \nu _2 \lambda _2^t + B_3(T) \nu _3 \lambda _3^t, \end{aligned}$$

where $\bar{A}^\mathcal {C}$, $\bar{A}^\mathcal {NC}$ and $\bar{s}$ denote the steady state values of $A_{t}^\mathcal {C}$, $A_{t}^\mathcal {NC}$ and $s_t$, $\lambda _i$ are the eigenvalues and $\nu _i$ the eigenvectors of the matrix *M*, and $B_i(T)$ are constants determined by the initial and terminal conditions of the stock and the emission abatement levels ($i=1,\dots ,3$).

Calculating the steady state values by setting

78 $$\begin{aligned} A_{t+1}^\mathcal {C}=A_{t}^\mathcal {C} = \bar{A}^\mathcal {C},\quad A_{t+1}^\mathcal {NC}=A_{t}^\mathcal {NC} = \bar{A}^\mathcal {NC}\ ,\quad s_{t+1} = s_t = \bar{s}, \end{aligned}$$

yields:

79a $$\begin{aligned} \bar{A}^\mathcal {C}&= \frac{x}{x+y}\mathcal {E} \end{aligned}$$

79b $$\begin{aligned} \bar{A}^\mathcal {NC}&= \frac{y}{x+y}\mathcal {E}\end{aligned}$$

79c $$\begin{aligned} \bar{s}&= \frac{1-\delta }{\delta } \frac{\mathcal {E}}{x+y} \end{aligned}$$

In addition, for the matrix *M* we derive the following eigenvalues $\lambda _i$ ($i=1,\dots ,3$):

80a $$\begin{aligned} \lambda _1&= \frac{1}{\delta }, \end{aligned}$$

80b $$\begin{aligned} \lambda _2&= \frac{1+\delta +\delta (x+y)-\sqrt{[1+\delta +\delta (x+y)]^2-4\delta }}{2\delta }\ , \end{aligned}$$

80c $$\begin{aligned} \lambda _3&= \frac{1+\delta +\delta (x+y)+\sqrt{[1+\delta +\delta (x+y)]^2-4\delta }}{2\delta }\ , \end{aligned}$$

and eigenvectors ($$i=1,\dots ,3$$):

81a $$\begin{aligned} \nu _1&= \left\{ -1,1,0\right\} ,\end{aligned}$$

81b $$\begin{aligned} \nu _2&= \left\{ \frac{x}{x+y}(1-\lambda _2), \frac{y}{x+y}(1-\lambda _2), 1 \right\} , \end{aligned}$$

81c $$\begin{aligned} \nu _3&= \left\{ \frac{x}{x+y}(1-\lambda _3), \frac{y}{x+y}(1-\lambda _3), 1 \right\} , \end{aligned}$$

Inserting into Eq. (77) yields:

82a $$\begin{aligned} A_{t}^\mathcal {C}&= \bar{A}^\mathcal {C} - B_1(T)\lambda _1^t + \frac{x}{x+y}\left[ B_2(T)(1-\lambda _2)\lambda _2^t + B_3(T)(1-\lambda _3) \lambda _3^t\right] \end{aligned}$$

82b $$\begin{aligned} A_{t}^\mathcal {NC}&= \bar{A}^\mathcal {NC} + B_1(T)\lambda _1^t + \frac{y}{x+y}\left[ B_2(T)(1-\lambda _2)\lambda _2^t + B_3(T)(1-\lambda _3) \lambda _3^t\right] \end{aligned}$$

82c $$\begin{aligned} s_{t}&= \bar{s} + B_2(T) \lambda _2^t + B_3(T) \lambda _3^t \end{aligned}$$

The constants $B_i(T)$ ($i=1,\dots ,3$) are derived from the initial stock $s_0$ of cumulative GHG emissions and the terminal conditions $A_{T}^\mathcal {C} = 0$ and $A_{T}^\mathcal {NC} = 0$ of aggregate emission abatement levels, which imply

83a $$\begin{aligned} B_1(T)&= \frac{y \bar{A}^C - x \bar{A}^{NC} }{(x+y)\lambda _1^T}, \end{aligned}$$

83b $$\begin{aligned} B_2(T)&= - \frac{\bar{A}^C + \bar{A}^{NC} + (s_0-\bar{s})(1-\lambda _3)\lambda _3^{T}}{(1-\lambda _2) \lambda _2^T - (1-\lambda _3) \lambda _3^T}, \end{aligned}$$

83c $$\begin{aligned} B_3(T)&= \frac{\bar{A}^C + \bar{A}^{NC} + (s_0-\bar{s})(1-\lambda _2)\lambda _2^{T}}{(1-\lambda _2) \lambda _2^T - (1-\lambda _3) \lambda _3^T}. \end{aligned}$$

By inserting these expressions back into equations () yields the aggregate abatement levels $A_t^\mathcal {C}$ and $A_t^\mathcal {NC}$ and the stock of aggregate cumulative emissions $s_t$ in the subgame perfect Nash equilibrium ($t=0,\dots ,T$).

Finally, we determine the individual countries’ abatement levels in the subgame perfect Nash equilibrium. Using backward induction starting from $t=T$, we obtain from equations ():

84a $$\begin{aligned} a_T^i&= 0 ,\qquad \forall \ i \in \mathcal {I}\ ,\end{aligned}$$

84b $$\begin{aligned} a_t^i&= \frac{A^\mathcal {C}_t}{\alpha _i \mathcal {A}^\mathcal {C}}, \qquad \forall \ i\in \mathcal {C}, \quad t=0,\dots ,T-1,\end{aligned}$$

84c $$\begin{aligned} a_t^i&= \frac{\gamma _i}{\Gamma ^\mathcal {NC}} A^\mathcal {NC}_{t}, \qquad \forall \ i\notin \mathcal {C}, \quad t=0,\dots ,T-1\ . \end{aligned}$$

$$\square$$

## Footnotes

1 In this protocol, the industrialized countries of the world, so-called “Annex B countries”, committed themselves to a reduction of greenhouse gas (GHG) emissions by 5.2% against 1990 levels over the period from 2008 to 2012.

2 Our model is a dynamic stock pollution game similar to Dockner and Van Long (1993), but generalized to *n* players, in discrete time. In addition, we use a carbon budget approach (see Sect. 2 for details), i.e., we abstract from stock depreciation.

3 Non-cooperative and cooperative approaches have been pursued. Many authors have stressed that the grand coalition is not stable if an individual defection does not destroy any coalition formation (e.g., Carraro and Siniscalco 1993; Eyckmans et al. 1993; Barrett 1994; Tol 1999; Bosetti et al. 2009.) Typically, in such circumstances, stable coalitions only contain a limited number of countries (see, e.g., Hoel 1991; Carraro and Siniscalco 1992; Finus and Caparrós 2015a, b). d’Aspremont et al. (1983) have conducted an original analysis and has introduced the definitions for internally and externally stable coalitions. Pioneering in the modelling of coalition structures are Bloch (1997) and Yi (1997).

4 In this respect our model shares some similarities with Harstad (2020), who analyzes a dynamic model inspired by the pledge-and-review mechanism of the Paris Agreement to account for a variety of different empirical observations of international environmental agreements.

5 If complete contracts on emission reductions could be written between countries, a first-best solution would be easily achieved including, of course, the initial participation problem. Harstad (2012, 2016) and Battaglini and Harstad (2016) analyze the interaction between decisions on emission levels and investments into low-carbon energy technologies in dynamic games with incomplete contracting, i.e., when countries can contract on emission reductions but not on investment. We abstract from technology investments and scrutinize what refunding can achieve in case countries cannot contract ex ante on emission reductions.

6 Dynamic games on voluntary provision of public goods have been significantly developed (see Wirl 1996; Dockner and Sorger 1996; Sorger 1998; Marx and Matthews 2000). Recent contributions involve Dutta and Radner (2009) who examine agreements on mitigating climate change supported by inefficient Markov perfect equilibria.

7 In this respect, our argument is exactly opposite to Gerber and Wichardt (2013), who propose to split static public good problems into dynamic ones in order to reduce downside payments.

8 Throughout the paper, we denote the time horizon by *T*. Note that a time horizon of *T* comprises of $T+1$ periods.

9 We do not restrict abatement to be at most as high as business-as-usual emissions, i.e., $a^i_t \le \epsilon _i$. Thus, we allow for negative net emissions, for example via afforestation or carbon capture and sequestration technologies.

10 This is a standard short-cut way of capturing aggregate abatement costs in country *i* (see, e.g., Falk and Mendelsohn 1993).

11 The equilibrium payoff $W_{t+1}^i(s_{t+1})$ is minus the discounted sum of the total domestic costs over the remaining time horizon starting from period $t+1$ in the subgame perfect equilibrium of the subgame starting in period $t+1$.

12 Despite being closed-form the solutions are quite cumbersome and, thus, relegated to the “Appendix”.

13 Both the global social optimum and the decentralized outcome are important benchmarks in evaluating the performance of potential international agreements. While the decentralized outcome is realized if no agreement takes place, the social optimum is the ultimate goal an international agreement seeks to implement. Obviously, any agreement has to outperform the decentralized outcome in order to be seriously considered, and it is the “better,” the closer its outcome is to the global social optimum.

14 While abatement paths with ${a_t^i}^\star < \tilde{a}_t^i$ would also be “feasible” in a strictly technical sense and could also be implemented by the RS, we consider the social global optimum as a natural upper bound for the emission abatement levels of coalition members.

15 The set of initial payments implicitly defines a transfer scheme $\mathcal {T}$, as introduced in Sect. 3.

16 The main obstacles for translating an upper temperature bound for mean global surface temperature into a carbon budget are scientific uncertainties concerning the equilibrium climate sensitivity and the climate-carbon cycle feedback (see, e.g., Friedlingstein et al. 2011; Zickfeld et al. 2009).

17 In particular, treaties with large coalition sizes and high aspired abatement paths may not be stable, as countries may have an incentive not to participate in the treaty in the first place in order to free-ride on the abatement efforts of the remaining coalition members.

18 We apply the usually made assumption that countries stick with the coalition in case of indifference.

19 For optimal transfer systems in the presence of asymmetries, see Finus and McGinty (2019).

20 The twelve regions are: United States of America (US), European Union (EU), Japan, Russia, Eurasia, China, India, Middle East (MidEast), Africa, Latin America (LatAm), other high income countries (OHI) and Rest of the World (Others).

21 As in the RICE-2010 model, we use ten year periods. However, we usually express all values in per annum terms.

22 In practice, these countries must be collectively stubborn and insist on full participation by this entire core group before going ahead.

23 Development-compatible refunding system have been developed in Gersbach and Hummel (2016).

24 However, whether a change in the exogenous parameters increases or decreases the necessary level of global fund depends, in most cases, on the whole set of exogenous parameters, and the comparative static results for the global fund of the respective RS are quite complex.

25 How public debt can be used to strike an intergenerational bargain in the context of climate change is also addressed by Dennig et al. (2015) who propose several focal bargaining points.

26 The Green Climate Fund was formally established during the UNFCCC COP-16 meeting in Cacun in 2010. Its objective is to assist developing countries in adaptation and mitigation practices to counter climate change.
